# European national health plans and the monitoring of online searches for information on diabetes mellitus in different European healthcare systems

**DOI:** 10.3389/fpubh.2022.1023404

**Published:** 2022-11-24

**Authors:** Irene Bosch-Frigola, Fernando Coca-Villalba, María José Pérez-Lacasta, Misericòrdia Carles-Lavila

**Affiliations:** ^1^Department of Economics, Rovira i Virgili University, Reus, Spain; ^2^Facultad de Comunicación y Ciencias Sociales, Universidad San Jorge, Zaragoza, Spain; ^3^Research Group on Statistics, Economic Evaluation and Health (GRAEES), Reus, Spain; ^4^Research Center on Economics and Sustainability (ECO-SOS), Reus, Spain

**Keywords:** diabetes mellitus, broken-line models, factor analysis of mixed data, Google trends, healthcare system

## Abstract

Diabetes mellitus (DM) is a serious non-communicable disease (NCD) and relies on the patient being aware of their condition, proactive, and having adequate medical care. European countries healthcare models are aware of the impact of these variables. This study evaluates the impact of online health information seeking behavior (OHISB) during World Diabetes Mellitus Day (WDMD) in European countries from 2014 to 2019 by grouping countries according to the changes in citizens' search behavior, diabetes mellitus prevalence, the existence of National Health Plans (NHP), and their respective healthcare systems. We extracted data from Global Burden of Disease, Google Trends (GT), Public Health European Commission, European Coalition for Diabetes, and the Spanish Ministry of Health. First, we used the broken-line models to analyze significant changes in search trends (GT) in European Union member countries in the 30-day intervals before and after the WDMD (November 14) from 2014 to 2019. Then the results obtained were used in the second phase to group these countries by factor analysis of mixed data (FAMD) using the prevalence of DM, the existence of NHP, and health models in each country. The calculations were processed using R software (gtrendsR, segmented, Factoextra, and FactoMineR). We established changes in search trends before and after WDMD, highlighting unevenness among European countries. However, significant changes were mostly observed among countries with NHP. These changes in search trends, in addition to being significant, were reiterated over time and occurred especially in countries belonging to the Beveridge Model (Portugal, Spain, and Sweden) and with NHPs in place. Greater awareness of diabetes mellitus among the population and continuous improvements in NHP can improve the patients' quality of life, thus impacting in disease management and healthcare expenditure.

## Introduction

One of Europe's most serious chronic metabolic disorders is diabetes mellitus (DM), and it is on the rise (1). DM affected approximately 463 million people in 2019 and is predicted to affect 578 million by 2030 and 700 million by 2045. However, it was also observed that its prevalence is higher in urban than rural areas (2). Worldwide, an estimated 19.3% of the population between 65 and 99 years live with this non-communicable disease (NCD) (3). Therefore, to achieve good control of this NCD, regardless of its etiological classification, it is necessary to follow exhaustively the guidelines provided by health professionals to have a good quality of life (4–6). Patient prescriptions should always be adapted to the reality of each case and include guidelines related to hypoglycemic medication and healthy lifestyles ranging from a balanced diet to regular physical exercise (7).

Patients and their environment must be aware of the critical nature of DM. In case of poor management, it can have serious consequences that can affect the body in the short- (e.g., most frequently severe hypoglycemia and hyperglycemia) or medium/long-term (e.g., diabetic nephropathy, diabetic neuropathy, diabetic retinopathy, etc.), which can be very serious and can cause death (8–10).

Health systems are the first lines of care for their citizens' health. Although the structure of these systems differs according to the models to which the European countries being analyzed belong, the underlying goal of them is to provide good care to the population in order to look after their health (11–15). There are numerous frameworks that could be used to categorize countries of the European Union into healthcare system typologies (16–20). One of them [(18), p. PE114], defines “Beveridge” Systems, “Bismarck” Systems and Mixed Systems. Amongst other differences between these models, the Bismarck Model finances healthcare through compulsory social security contributions by employees and employers, while the Beveridge model was financed through public taxes. Further, while the Beveridge model is referred to as National Health System, those under the Bismarck model is also referred as Social Health Insurance System. The Mixed model is a different categorization. It's also referred as the Private Health Insurance System.

According to Gaeta et al. [(18), p. E114], these are the European countries following each healthcare system: Bismarck model, Belgium, Czech Republic, Estonia, France, Germany, Hungary, Lithuania, Luxembourg, Netherlands, Poland, Romania, Slovakia, and Slovenia; Beveridge model, Cyprus, Denmark, Finland, Ireland, Italy, Latvia, Malta, Portugal, Spain, Sweden, and United Kingdom; Mixed model: Austria, Bulgaria, Croatia and Greece.

The governments of different countries, public and private organizations, researchers, and healthcare providers are aware of the concerning rise in the number of cases of DM and the related work carried out jointly by the different international organizations. Therefore, they have promoted public policies that have boosted prevention campaigns aimed at the population, encouraging diabetes education, early detection, and promoting healthy lifestyles. These actions aim to raise public awareness of this condition and enable patients to adequately manage the acute symptoms of their chronic condition (1, 2, 21, 22). Additionally, the National Health Plans (NHP) have been established recently, including initiatives targeted toward DM (23, 24). According to a past study (23), the European countries of Austria, Bulgaria, Croatia, Czechia, Denmark, Finland, Greece, Hungary, Ireland, Italy, Netherlands, Poland, Portugal, Romania, Slovakia, Slovenia, Spain, and Sweden would have implemented NHPs. On the other hand, Belgium, Cyprus, Estonia, France, Germany, Latvia, Lithuania, Luxembourg, Malta, and United Kingdom would not have implemented NHPs or would be in the process of implementation.

Patients can use online resources and platforms to promote their well-being and improve that of those around them (25–28). For example, they can use online resources when suffering from a disease, its complications, having an interest in taking care of their health, or to simply be informed and seek real-time answers (29, 30). It should be noted that healthcare providers and official sources can bring the most accurate information as they know the pathology concerned (31). However, suppose users choose to search through the current online resources offered through the Internet (as a public information provider). In that case, such interests of citizens can be monitored through search engine data. Google (32) is one of the relevant search engines, being the most widely used worldwide and having a large market share (33). In this regard, Google Trends (GT) (34) is becoming a tool that is raising special interest for probable forecasts and in understanding the interests of the population (35). Data provided by GT can be used as a “surrogate” of online health information seeking behavior (OHISB) and give useful information on search behaviors related to health or healthy habits (33, 36–38). Some research examines the association between data provided by internet sources and public health policies (39).

Within this framework and as evidenced in previous research papers (40–45), global days dedicated to raising public awareness of specific health issues can lead to Internet search by citizens and provide valuable information on search behaviors. In particular, under the initiative of the International Diabetes Federation and with the support of World Health Organization, November 14 was established as the world DM day (WDMD) (46). This initiative was an attempt to promote information related to the treatment, prevention, and timely diagnosis, as well as raise awareness of DM among the population. It should be emphasized that DM evokes serious impacts at both the human and economic dimensions. Therefore, based on this information and considering the impact of DM on the current healthcare systems, this original research analyzes the prevalence of this disease in Europe. We used the European healthcare system model (Bismarck, Beveridge and mixed models) of countries to monitor the interest raised in the European population on the WDMD and 30 days before and after it, using two different methodologies to reach the output.

This study evaluates the impact of World Diabetes Mellitus Day (WDMD) in European countries from 2014 to 2019, grouping countries according to the changes in the citizens' search behaviors by the prevalence of the disease, the existence of NHP, and their respective healthcare systems. The period of this study began in 2014, when a prior study analyzing the NHP in Europe (23) was published. The period under analysis extends to 2019, namely, before pre-pandemic trends.

This research paper is structured as follows: Section 2 (materials and methods) explains the steps to set up the Database (DB) as well as the methodology and software used. Section 3 (results) shows the results of the research, and Section 4 (discussion) discusses the relevant findings of the present work.

## Materials and methods

The research methodology is structured in the following steps: DB approach and sources consulted, and Methodology and software used.

### First step: Database approach and sources consulted

#### Prevalence of DM

Using the data provided by the Global Burden of Disease Collaborative Network-Global Burden of Disease Study (47), it was feasible to set up the database based on the information related to the prevalence of DM.

#### Monitoring of the population's interest

The Internet is an excellent environment to analyze the users' interests 30 days before and after November 14 (WDMD). The tool used in this case is GT on the European scale, used as an index of the population's interest related to DM. The information set by Relative Search Volumes (RSV) for the search term “Diabetes Mellitus” shows the proportion of queries for this centralized term in a specific time and region. Its standardization structured from 0 to 100 has been linked to the highest proportion of the searched term in each set year (34).

#### Official sources

The characteristics of healthcare systems in European Union countries have been analyzed based on different official sources from several organizations. The data have been retrieved from the “Public Health European Commission” (48), “Spanish Ministry of Health” (49), and “Health Care Systems in EU-28, National Health Care Service and Social Security System” (15).

#### European Coalition for Diabetes (EURADIA, FEND, International Diabetes Federation, and PCDE)

Information related to NHP existing in European countries has been compiled (23).

### Second step: Methodology and software

#### Methodology

Two methods were combined to achieve the required outputs of this novel research. First, to process the previously generated databases related to monitoring and search trends, it was necessary to use the broken-line models (BLM). With them, it was possible to track the population's interest during the 30 days before and after the WDMD (50–54). Once this output was achieved and processed to obtain the monitoring data, multivariate analysis was used (55–57). In particular, the factorial analysis of mixed data (FAMD) was used to classify the quantitative and qualitative variables of this research. The combination of these methods provided a major advantage in processing, visualizing, and analyzing the data set.

##### Broken-line models

Regression analysis analyzes dependent variables as a linear function of explanatory variables. In this framework, the model's response is presented as a linear function of the explanatory variables (58). However, the relationship between the response and some explanatory variables may not be linear. BLM are known to be segmented, and are represented using two or more straight lines; these lines are then connected at unknown values between the response and explanatory variables (53, 54). Here, it can be observed how the effect on the expected response of the breakpoints changes sharply. When this happens, it is necessary to use additional, non-standard optimization techniques to estimate the models because, in the estimation of the parameters of the cut-off points, the log-likelihood differs by segments, and the conditions established in the classical models are no longer fulfilled (50–54). According to Muggeo (52, 53) a segmented relationship is modeled when μ = *E*[*Y*] and variable Z is *i* = 1,2,3… *n* then


$$\begin{array}{l} \beta_{1}z_{i}+\beta_{2}{\left(z_{i}-\psi \right)}_{+}\\ \beta_{1}:left\;slope\\ \beta_{2}:difference-in-\;slopes\\ \psi :breakpoint \end{array}$$

Where (_*z*_*i*_−ψ)+_ = (*z*_*i*_−ψ) × *I*(*z*_*i*_>ψ) *and I*(·)

When a linear predictor is needed,


$$\begin{array}{l} \beta_{1}z_{i}+\beta_{2}{\left(z_{i}-\tilde{\psi }\right)}_{+}+\gamma I{\left(z_{i}>\tilde{\psi }\right)}^{-}\\ where\;I{\left(\cdot \right)}^{-}=-I\left(\cdot \right) \end{array}$$

At each iteration, the breakpoint value has to be updated,


$$\begin{array}{l} \tilde{\psi }=\tilde{\psi }+\hat{\gamma }/{\hat{\beta }}_{2} \end{array}$$

because a standard linear model must be fitted.

The Delta method for $\hat{\frac{\gamma }{\beta_{2}}}$ is obtained due to the standard error of $\tilde{\psi }$ when the algorithm converges.

Davies test (DT)

When breakpoint does not exist, the test for ψ is,


$$\begin{array}{l} H_{0}:\beta_{2}\left(\psi \right)=0 \end{array}$$

and there is no difference-in-slopes parameter. It is zero. β_2_ depends on a nuisance parameter, and ψ fades under H_0_. The DT (50) is useful for performing this hypothesis test. It must be noted that statistical tests such as Wald are not satisfied, and *p*-values are underestimated. Here, it must be pointed out that while DT is not suitable for selecting the number of the joinpoints, it is good for testing a breakpoint.

DT is framed in the range of Z when given the K fixed ordered values of breakpoints ψ_1_ < ψ_2_ < …ψ_*k*_.

When the standard normal distribution ψ_*k*_ is fixed, there is a relevant K of the test statistic {*S*(_ψ_*k*_)}*k* = 1, …, *K*_.

The one-side hypothesis test is,


$$\begin{array}{l} p-value\approx \Phi \left(-M\right)+Vexp\left\{{-M}^{2}/2\right\}{\left(8\pi \right)}^{-\frac{1}{2}} \end{array}$$

and the respective alternative is,


$$\begin{array}{l} H_{1}:\beta_{2}\left(\psi \right)>0. \end{array}$$

Being that the maximum of the K statistic *M* = max{*S*(_ψ_*k*_)}*k*_, Φ(·) is a function of standard normal,


$$\begin{array}{l} V=\sum_{k}\left(\left|S\left(\psi_{k}\right)-S\left(\psi_{k-1}\right)\right|\right) \end{array}$$

{*S*(_ψ_*k*_)}*k*_ is total variation.

##### Factorial analysis of mixed data

As aforementioned, the database used included qualitative and quantitative variables. The FAMD technique was employed because it allows both types of variables to be used simultaneously, generating a space of smaller dimensions. The quantitative variables were normalized, and the qualitative variables were treated in a normalized data table to balance all the variables under study. Principal component analysis and multiple correspondence analysis (59, 60) were combined to visualize the differences and the similarities (distances) of the analyzed elements, thereby helping us find the correlation of the continuous variables.

According to Pagès [(60), p. 71], there are two types of relationships:

a) From *R*^*K*^ toward *R*^1^.

Case 1) A quantitative variable:


$$\begin{array}{l} G_{s}\left(k\right)=\frac{1}{\sqrt{\lambda_{s}}}\underset{i}{\sum }p_{i}x_{ik}F_{s}\left(i\right)=r\left(k,F_{s}\right) \end{array}$$

Case 2) A category *k*_*q*_ of variable q: *F*_*s*_(*k*_*q*_) is the coordinate of the center of gravity of individuals with category (*k*_*q*_):


$$\begin{array}{l} G_{s}\left(k_{q}\right)=\frac{1}{\sqrt{\lambda_{s}}}\frac{1}{p_{k_{q}}}\underset{i}{\sum }p_{i}y_{ik_{q}}F_{s}\left(i\right)=\frac{1}{\sqrt{\lambda_{s}}}F_{s}\left(k_{q}\right) \end{array}$$

b) From *R*^*I*^ toward *R*^*K*^

It is expressed as follows:


$$\begin{array}{l} F_{s}\left(i\right)=\frac{1}{\sqrt{\lambda_{s}}}\underset{k\in K_{1}}{\sum }x_{ik}G_{s}\left(k\right)+\frac{1}{\sqrt{\lambda_{s}}}\underset{k_{q}\in K_{2}}{\sum }p_{k_{q}}\left(\frac{y_{ik_{q}}}{p_{k_{q}}}-1\right)G_{s}\left(k_{q}\right) \end{array}$$

#### Software used

We used open-source software libraries to obtain the results based on the abovementioned methodologies (53, 56, 59–62); specifically, we used gtrendsR and Segmented package for the BLM and FactoMineR and FactoExtra package for FAMD.

## Results

We obtained the results needed to develop this study with the DB created and previously mentioned techniques. Table 1 shows the variables related to the prevalence of DM and RSV from 2014–2019 for the European countries studied.

**Table 1:** Descriptive statistics for diabetes mellitus prevalence and relative search volumes from 2014–2019 for European countries.

	**Prevalence.14**	**Prevalence.15**	**Prevalence.16**	**Prevalence.17**	**Prevalence.18**	**Prevalence.19**
Mean	8172.12	8322.02	8495.52	8685.28	8932.79	9263.45
Variance	5120200.86	5384009.04	5599445.51	5852454.75	6274603.12	6907150.99
Standard deviation	2262.79	2320.35	2366.31	2419.18	2504.92	2628.15
Skewness	0.38	0.45	0.48	0.49	0.49	0.48
Kurtosis	2.48	2.63	2.68	2.70	2.71	2.72
Minimun	4525.27	4639.83	4696.55	4746.50	4855.84	5011.13
Maximun	13275.10	13735.64	14125.13	14509.00	15017.54	15686.45
Range	8749.83	9095.81	9428.59	9762.50	10161.70	10675.32
1st quartile	6305.72	6311.38	6422.73	6539.79	6708.08	6915.11
3rd quartile	9690.07	9692.30	9724.28	9975.68	10299.98	10712.67
Interquartile range	3384.35	3380.92	3301.55	3435.89	3591.90	3797.56
	**rsv.14**	**rsv.15**	**rsv.16**	**rsv.17**	**rsv.18**	**rsv.19**
Mean	23.91	23.66	20.80	22.63	23.41	23.50
Variance	567.20	589.35	420.58	549.92	395.04	525.93
Standard deviation	23.82	24.28	20.51	23.45	19.88	22.93
Skewness	0.47	0.43	0.06	0.42	−0.04	0.52
Kurtosis	2.33	1.67	1.09	1.62	1.43	2.28
Minimun	0.00	0.00	0.00	0.00	0.00	0.00
Maximun	80.50	66.50	49.50	61.50	58.00	74.50
Range	80.50	66.50	49.50	61.50	58.00	74.50
1st quartile	0.00	0.00	0.00	0.00	0.00	0.00
3rd quartile	39.00	42.50	40.50	39.00	42.00	36.00
Interquartile range	39.00	42.50	40.50	39.00	42.00	36.00

The results of BLM applied in the different European countries are shown in Annex.

**Figure 1:**
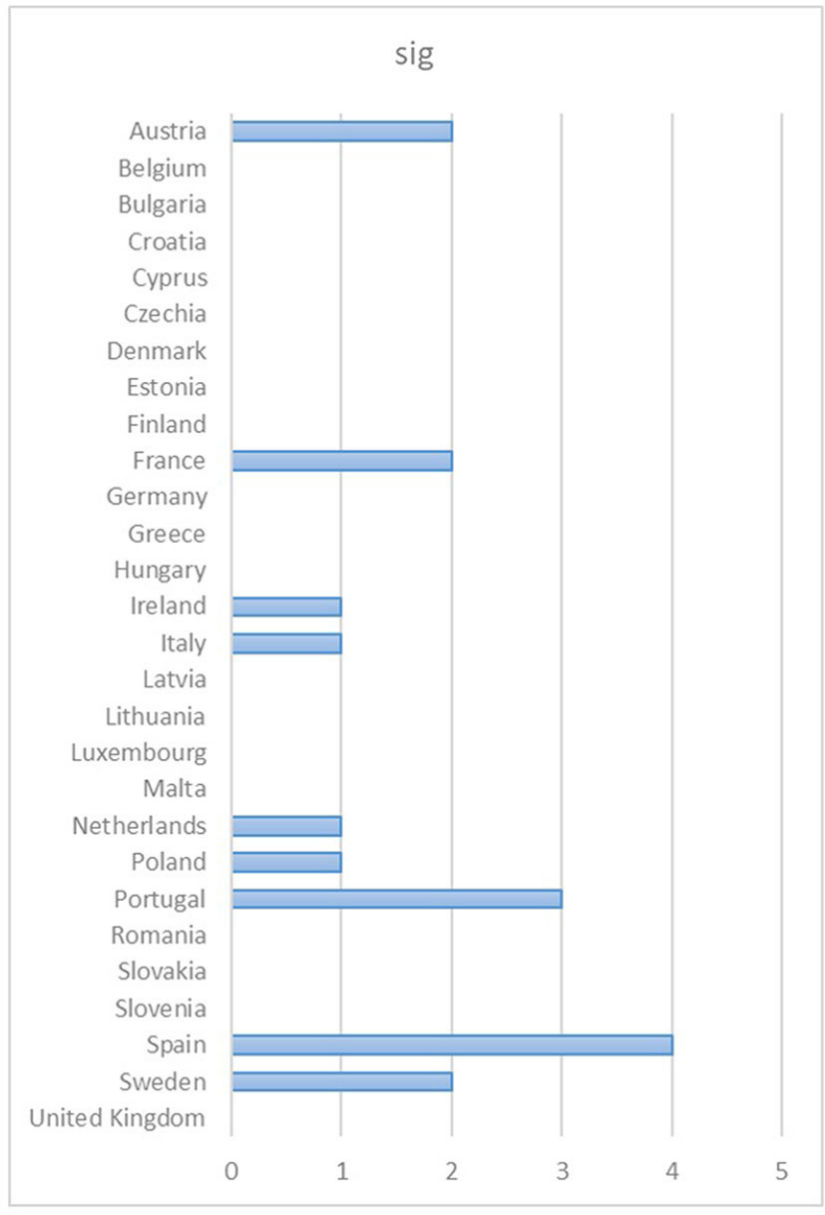
Changes in search trends on the designated day in the different European countries. Source: Compiled by the authors with data extracted from GT (2022).

The analysis of monitoring achieved through the BLM during the 30 days before and after WDMD was related to the change in the search trend on the designated day in the different European countries during the analyzed period. This variable is denoted as “sig” (Figure 1).

**Figure 2:**
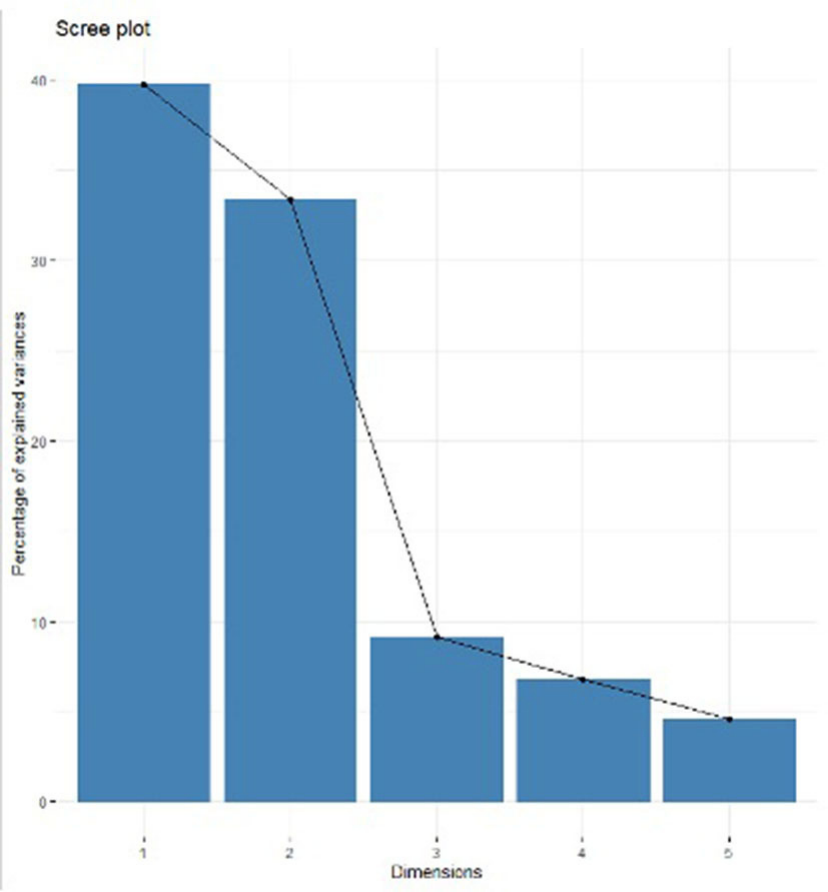
Percentages of inertia explained by each factorial analysis of mixed data dimension.

The multivariate analysis (Figure 2) showed that dimension 1 and 2 of the sedimentation graph accounted for 73.15% of the accumulated variance, whereas dimensions 3 and 4 accounted for 9.13 and 6.79% of the variance, respectively.

**Figure 3:**
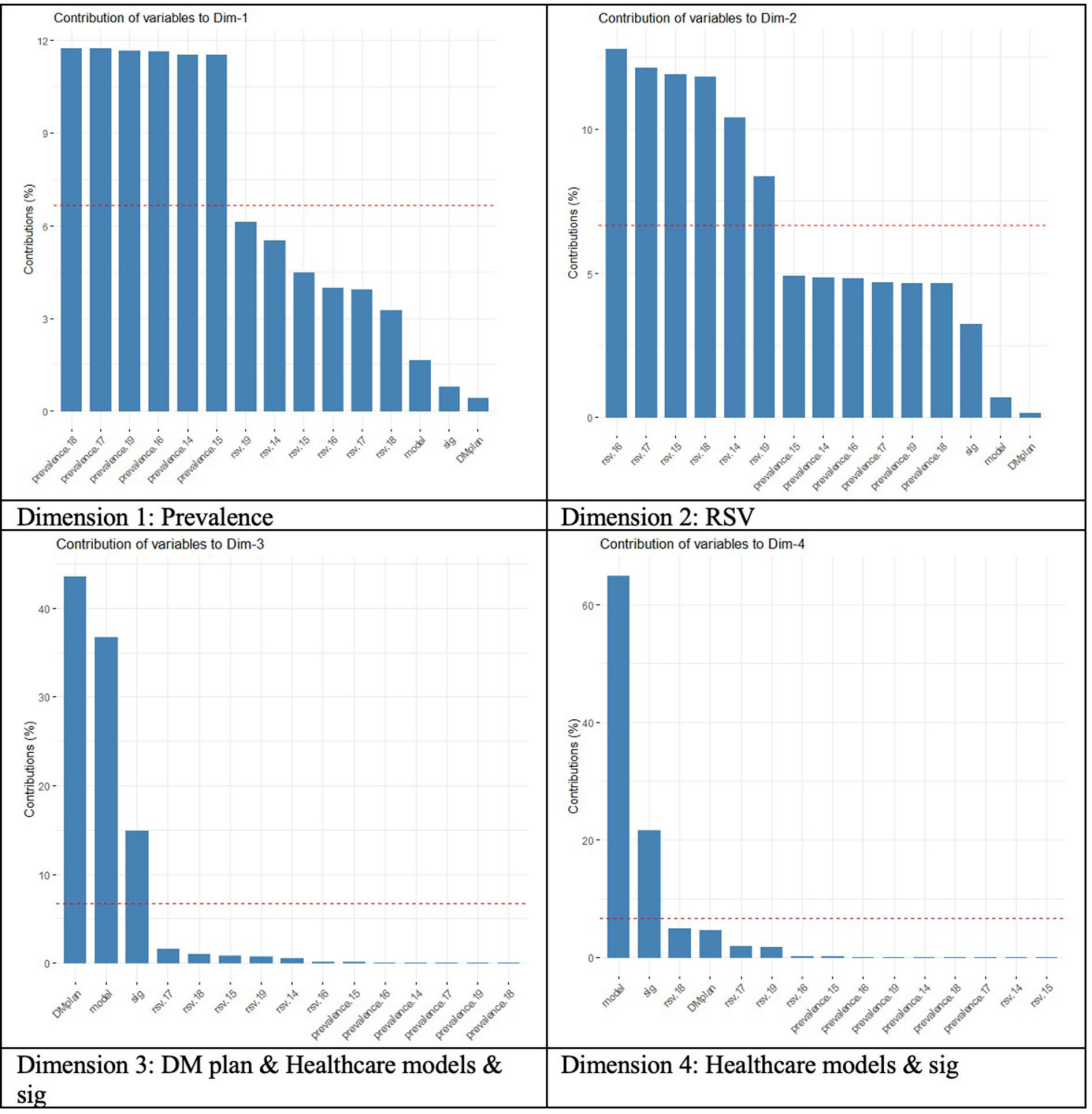
Contribution of variables to the principal dimensions (“Prevalence,” “RSV diabetes,” “DM day,” “DM plan, Healthcare models & Sig” and “Healthcare models & Sig”).

Figure 3 shows the four dimensions grouping the study variables. Dimension 1 shows the prevalence of DM. Dimension 2 shows the RSV related to DM. Dimension 3 shows the European countries analyzed that have a NHP (DM plan & Healthcare models & sig). Please, be noted that we coded this variable as “DM plan” Finally, Dimension 4 shows the monitoring analysis achieved when the BLM methodology was used 30 days before and after WDMD, getting the “sig” variable (explained above).

**Figure 4:**
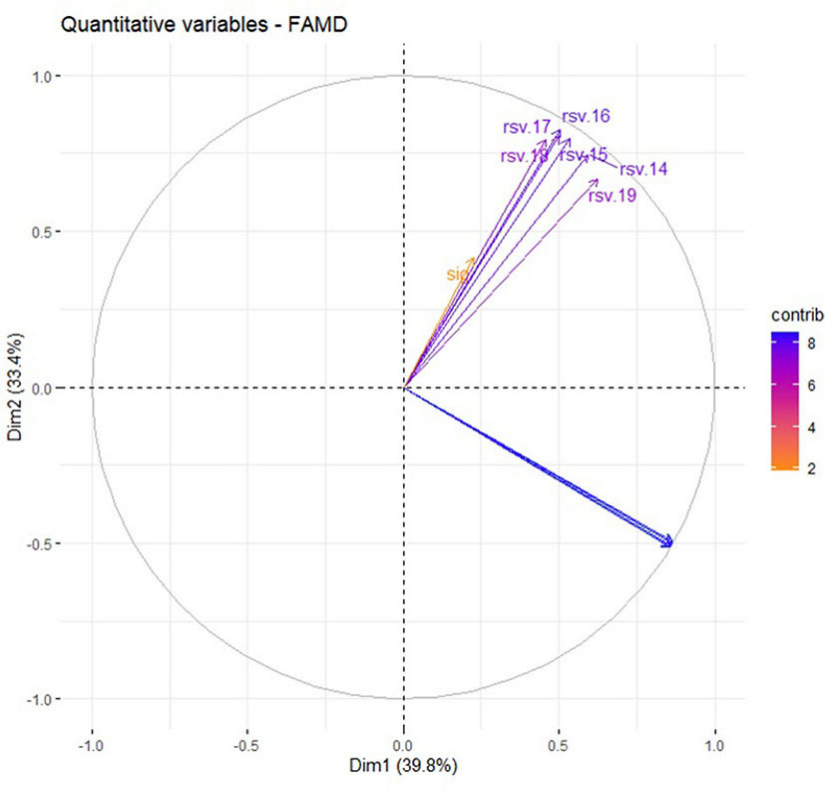
Relationship between variables and their contribution to dimensions 1 and 2.

**Figure 5:**
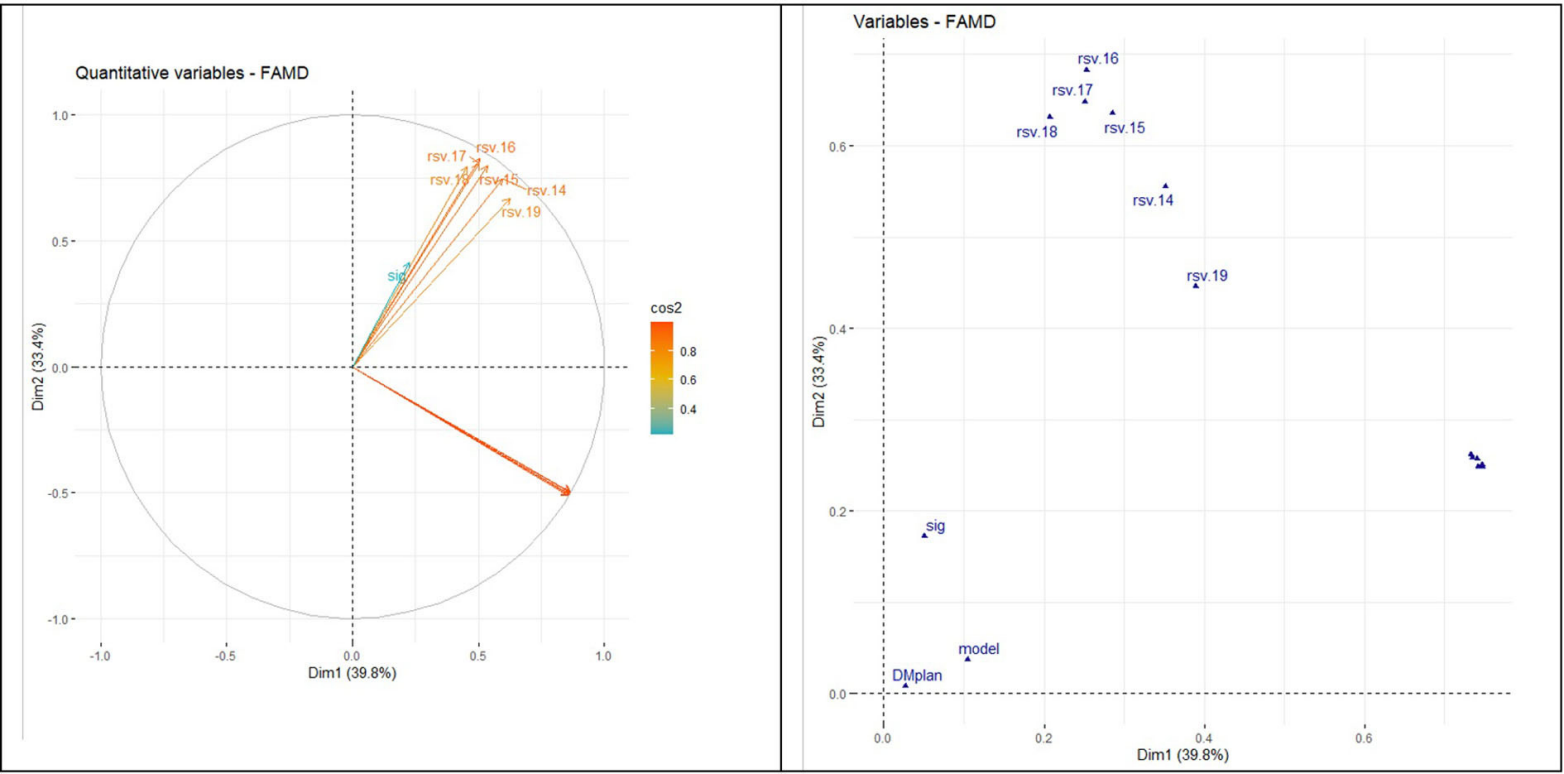
Relationship between quantitative and qualitative variables (dimensions 1 and 2).

Likewise, the correlation circle (Figures 4, 5) shows the relationship between quantitative variables and their contribution to dimensions 1 and 2. There is no correlation between RSV and prevalence of DM, but there are high contributions to dimensions 1 and 2, respectively.

**Figure 6:**
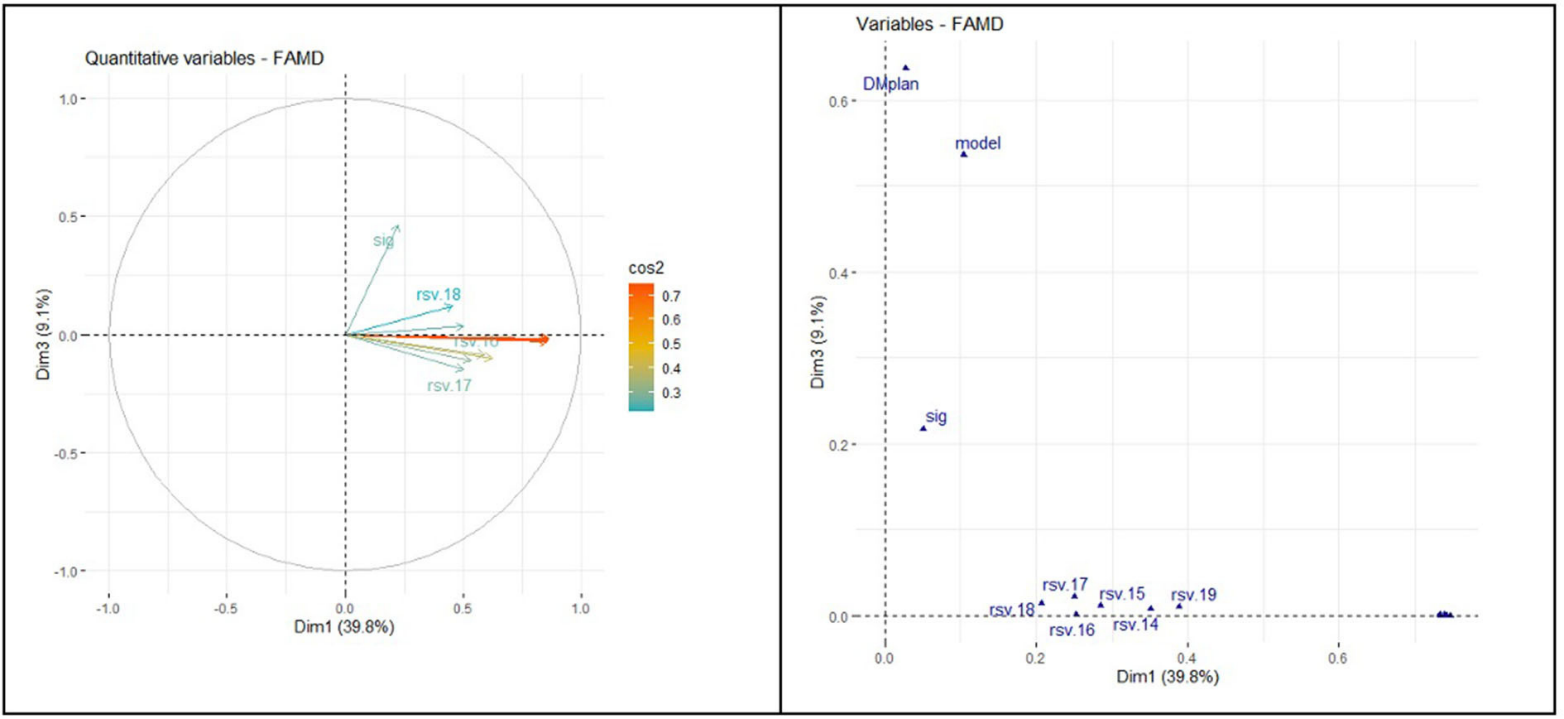
Relationship between quantitative and qualitative variables (dimensions 1 and 3).

The correlation circle in Figure 6 shows the relationship between the quantitative variables and their contribution to the factor map in dimensions 1 and 3. Prevalence of DM was unrelated to searches in the WDMD.

**Figure 7:**
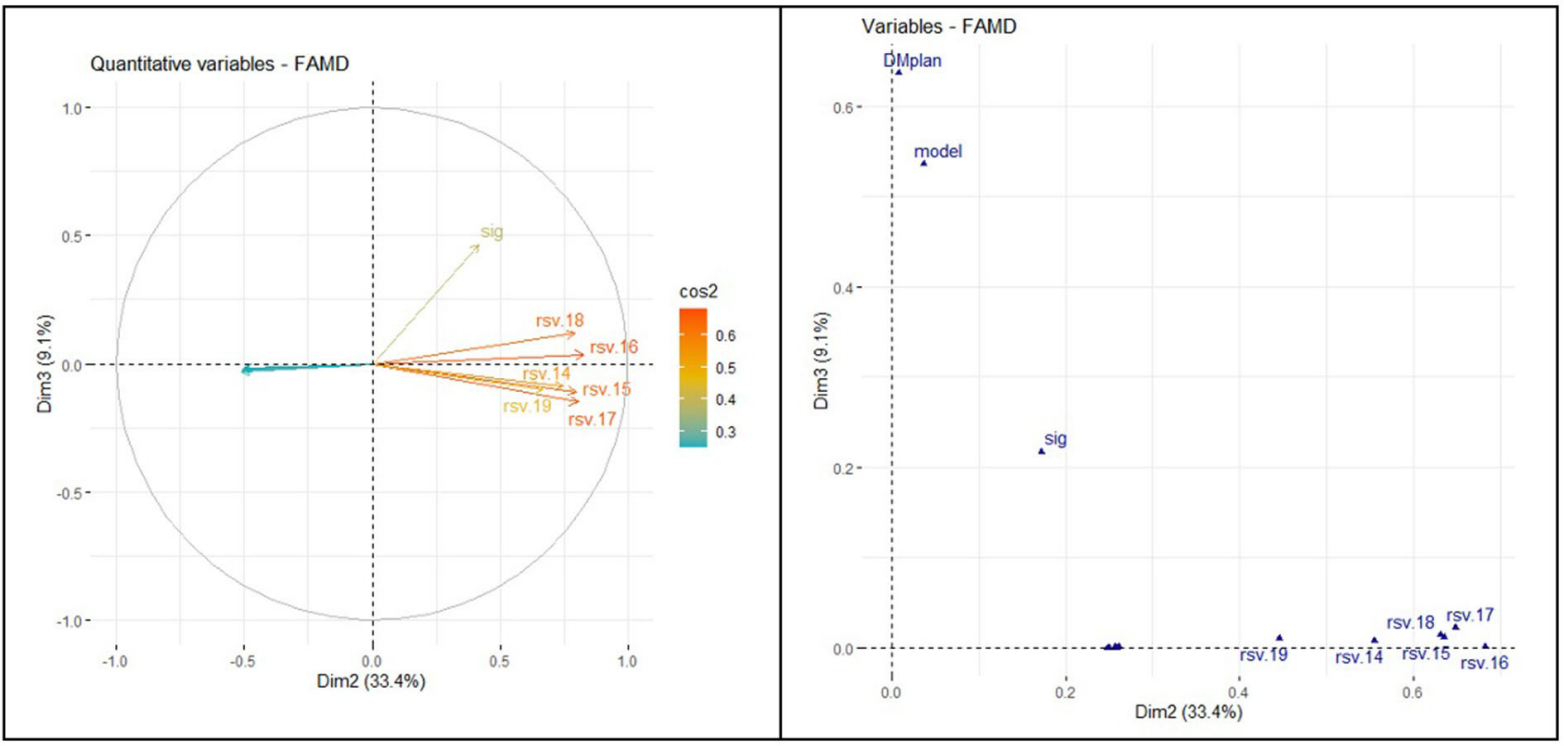
Relationship between quantitative and qualitative variables (dimensions 2 and 3).

The correlation circle in Figure 7 shows the relationship between the variable's contribution to the factor map in dimensions 2 and 3.

**Figure 8:**
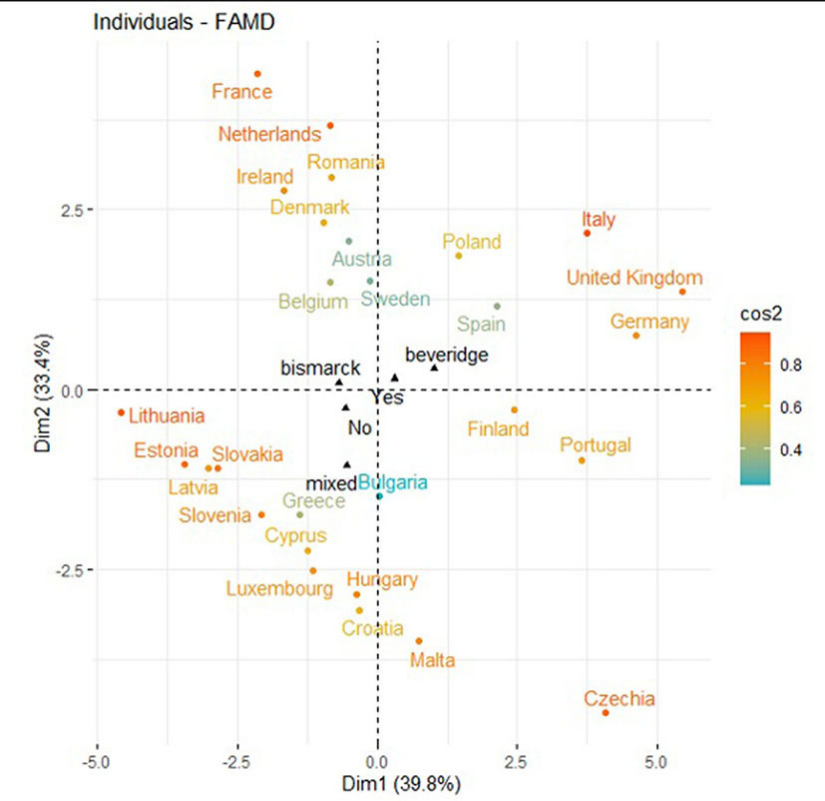
Distribution of the countries between dimension 1 and dimension 2.

**Figure 9:**
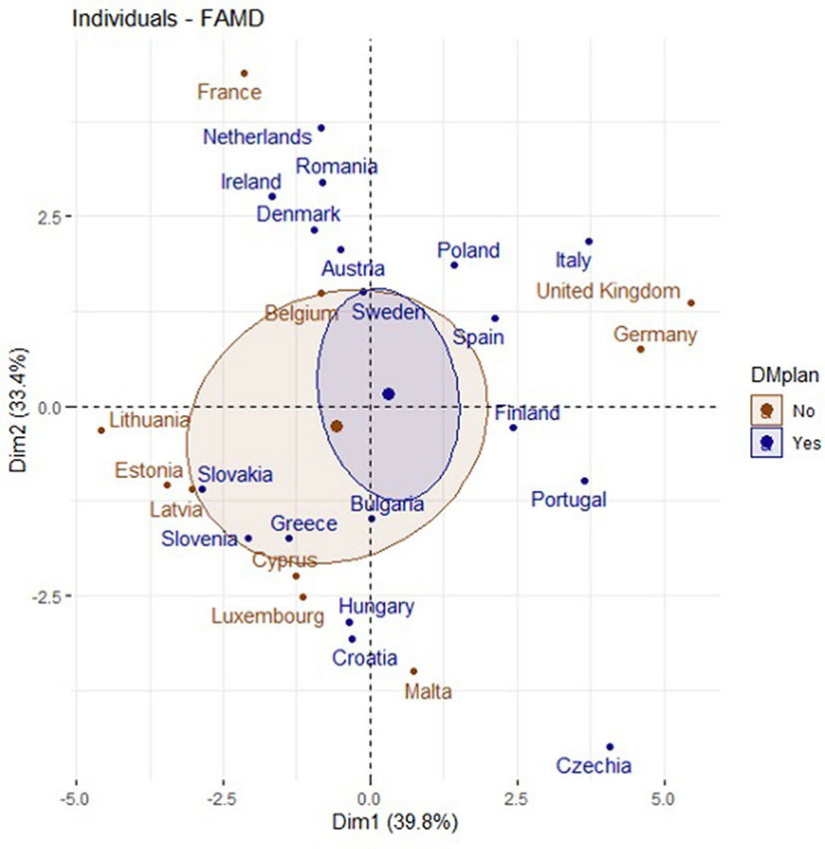
Countries with a NHPs and dimension 1 and dimension 2.

Figures 8, 9 show the distribution of the countries between dimension 1 and dimension 2. It can be noted that countries with a NHP and with high searches for the term “diabetes mellitus” had a high and low prevalence of this disease.

**Figure 10:**
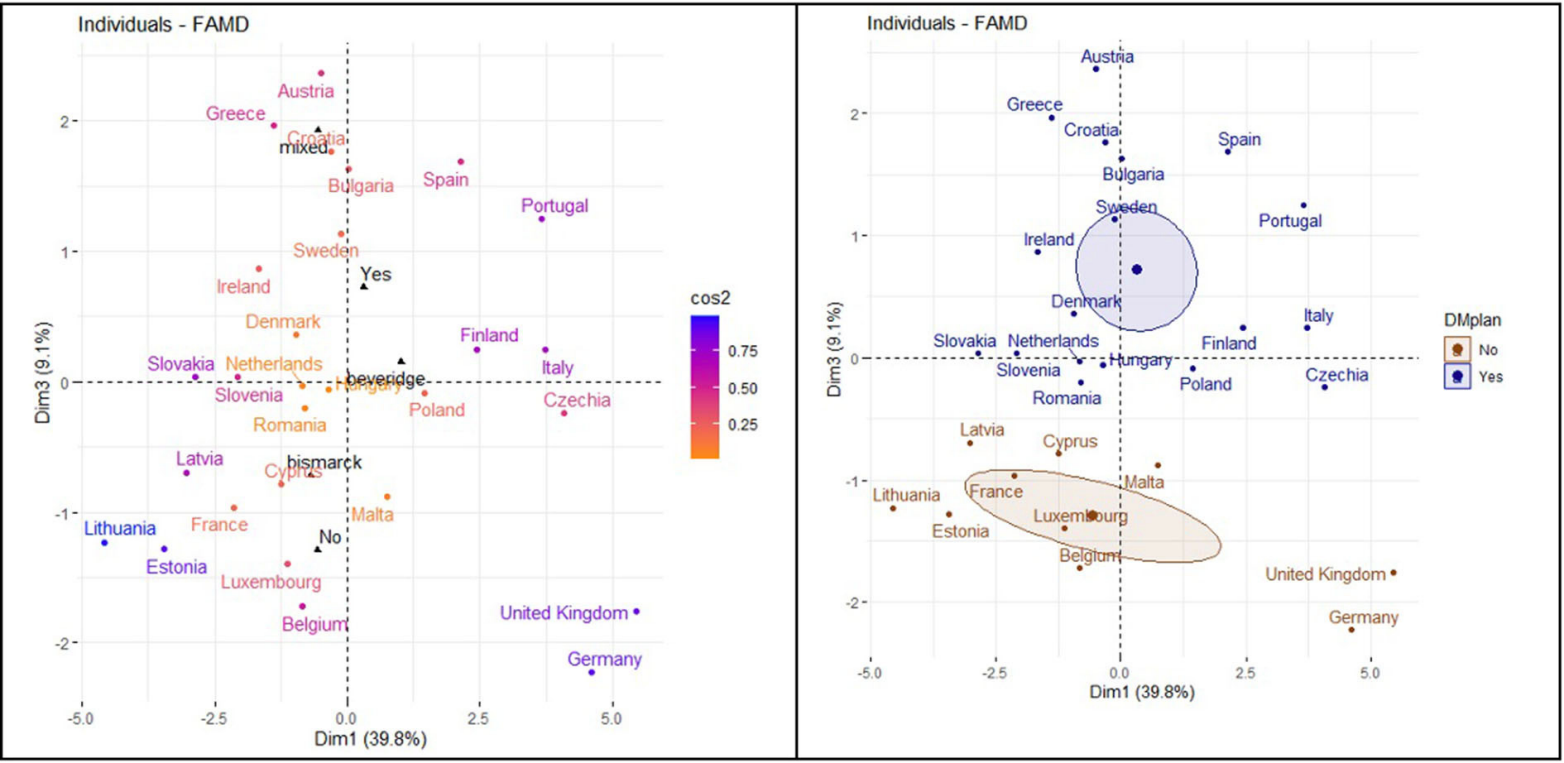
Distribution of the countries and dimension 1 and dimension 3.

Figure 10 shows the distribution of the countries in the factor map represented through dimension 1 and 3. Among countries with NHPs and higher prevalence of DM, many changes in internet search trends happened on WDMD.

**Figure 11:**
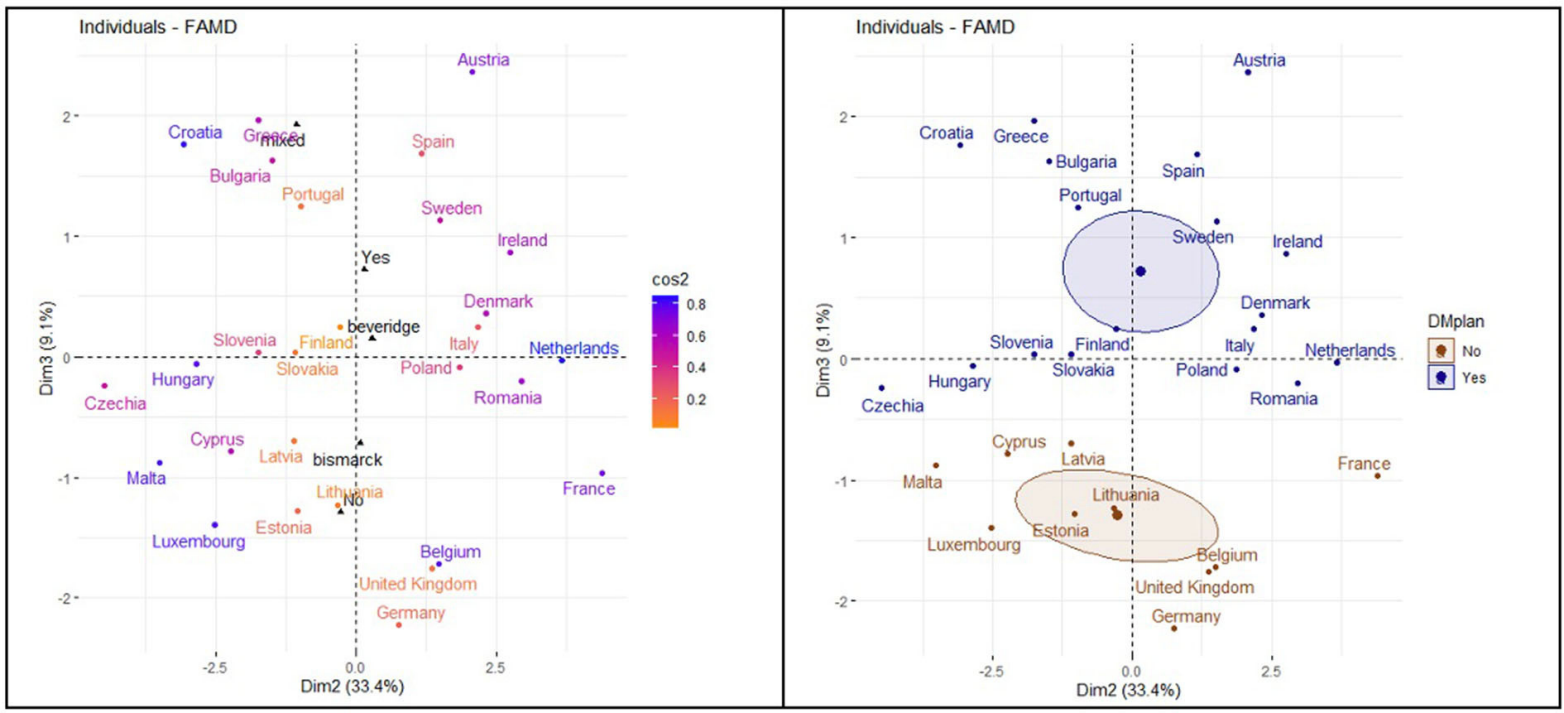
Distribution of the countries in dimension 2 and dimension 3.

The Figure 11 factor map is generated from dimension 2 and 3. Among countries with NHPs, higher searches for RSV imply a higher number of times of noticeable changes in internet searches on DM during the WDMD.

Consequently, changes in the search trend before and after the WDMD were uneven among European countries during the period analyzed. The most important changes were mostly observed among countries with NHPs. These noticeable changes in the search were reiterated over time and occurred especially in the countries belonging to the Beveridge model (Portugal, Sweden and Spain).

**Figure 12:**
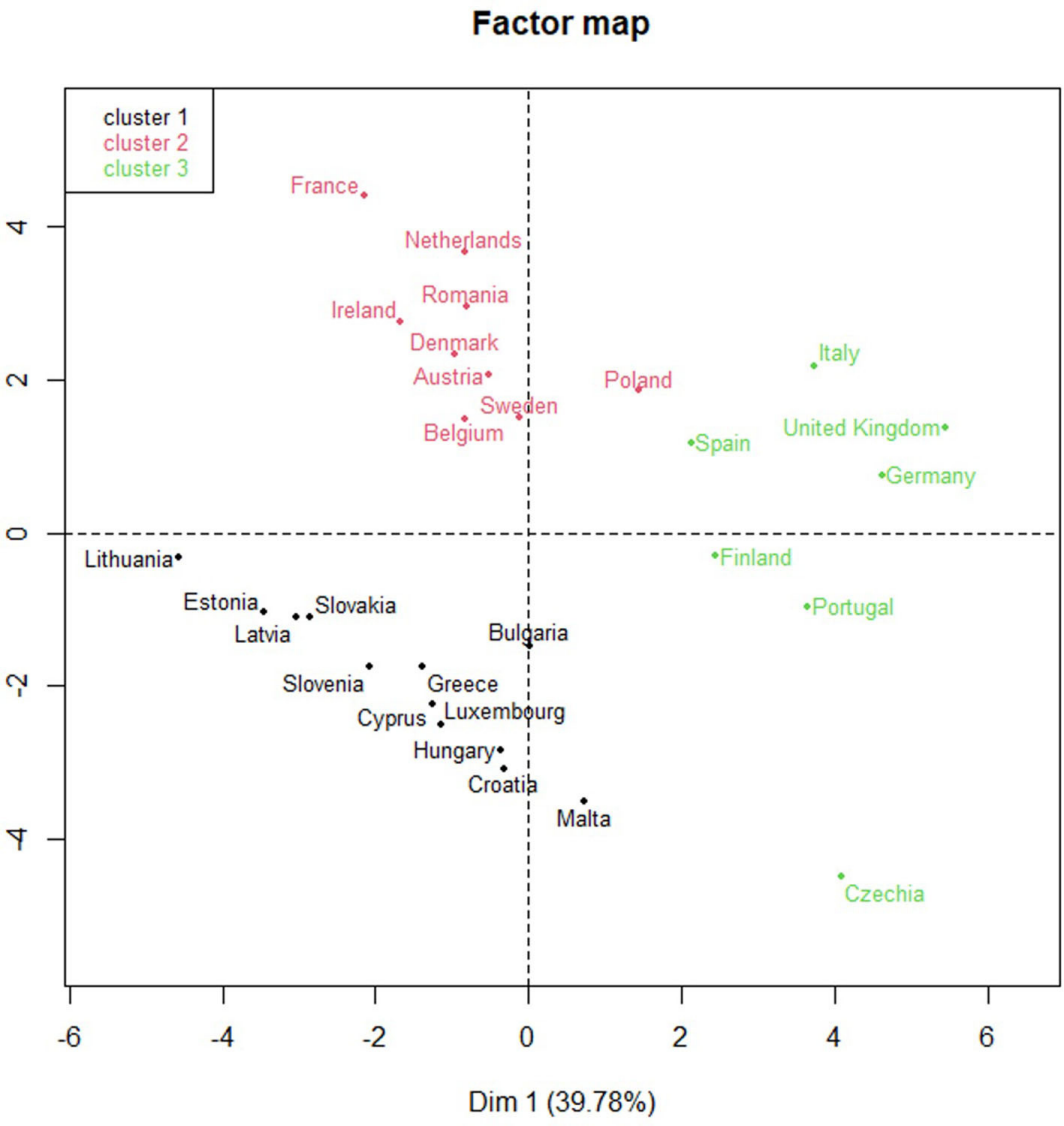
Classification of European countries by three clusters.

Figure 12 shows the classification obtained through the FAMD method of the European countries analyzed. The three clusters show the following coincidences:

Cluster 1 shows a comparatively low prevalence of DM, low RSV related to DM, half of the country samples with no NHP, and no considerable changes in internet search trends during the WDMD.
Cluster 2 shows, for the most part, a low prevalence of DM, a high RSV related to DM, countries with NHPs, and unevenly noticeable changes in internet search trends during the WDMD.
Cluster 3 shows a high prevalence of DM, a high RSV related to DM, and, for the most part, countries with NHPs. Rarely, they present significant changes in internet search trends during the WDMD.

**Figure 13:**
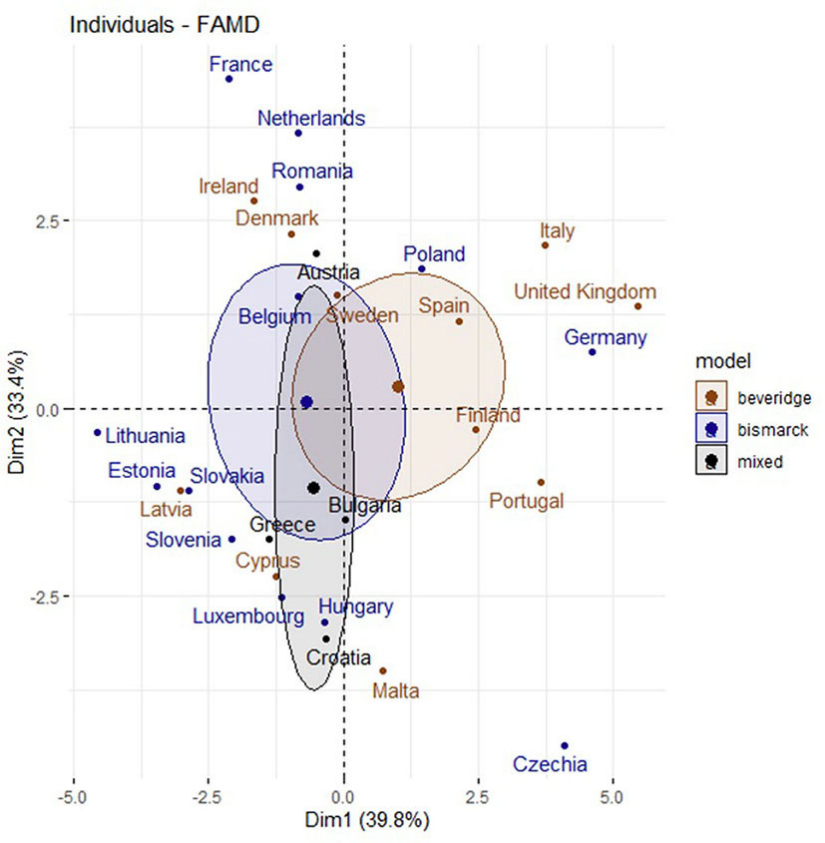
Distribution of the countries and dimensión 1 and dimensión 2.

Table 2 and Figure 13 shows, for each European country, the number of times that the search changes were significant and their respective clusters, the healthcare systems, and whether they have NHPs.

**Table 2:** Changes in citizens' search behavior, clusters, and healthcare systems.

**Country**	**Model**	**DMplan**	**Sig**	**Cluster**
Austria	Mixed	Yes	2	2
Belgium	Bismarck	No	0	2
Bulgaria	Mixed	Yes	0	1
Croatia	Mixed	Yes	0	1
Cyprus	Beveridge	No	0	1
Czechia	Bismarck	Yes	0	3
Denmark	Beveridge	Yes	0	2
Estonia	Bismarck	No	0	1
Finland	Beveridge	Yes	0	3
France	Bismarck	No	2	2
Germany	Bismarck	No	0	3
Greece	Mixed	Yes	0	1
Hungary	Bismarck	Yes	0	1
Ireland	Beveridge	Yes	1	2
Italy	Beveridge	Yes	1	3
Latvia	Beveridge	No	0	1
Lithuania	Bismarck	No	0	1
Luxembourg	Bismarck	No	0	1
Malta	Beveridge	No	0	1
Netherlands	Bismarck	Yes	1	2
Poland	Bismarck	Yes	1	2
Portugal	Beveridge	Yes	3	3
Romania	Bismarck	Yes	0	2
Slovakia	Bismarck	Yes	0	1
Slovenia	Bismarck	Yes	0	1
Spain	Beveridge	Yes	4	3
Sweden	Beveridge	Yes	2	2
United Kingdom	Beveridge	No	0	3

Therefore, the combination of the results using two methods (BLM and FAMD) leads to the following patterns: a high RSV related to DM and higher prevalence of DM was noted in countries with NHPs, which showed a greater number of considerable changes in search trends during the WDMD. These changes in OHISB were reiterated over time and happened mainly in countries with NHPs and belonging to the Beveridge Model (Portugal, Spain, and Sweden). Meanwhile, most countries belonging to the Bismarck model, with NHPs, and a high frequency of searches for DM showed a low prevalence of DM compared to the others. Therefore, they have not presented noticeable changes in search trends during the WDMD.

## Discussion

Our research work demonstrates the potential of analyzing user activity collected through Google and GT. Our results concur with those of prior research (33, 36, 40, 43), and it emphasizes the importance of OHISB evaluation in current days for raising public awareness of specific health issues (37, 40–45). Furthermore, our study is novel as it evaluates the impact of OHISB on the WDMD considering selected European countries and depending on whether they have NPHs or not, their healthcare system model, and the prevalence of DM among the population. We have managed to extract the respective patterns framed between 2014 and 2019. We emphasize that the information related to the NHPs of each European country, used to classify them, was extracted from the (23), as shown in section 1 introduction of this study. Two groups were created: whether they were implemented or not (countries that were in the process of establishing the NPHs were classified as “not implemented” as they were in the process of doing so). Please, note that numerous frameworks could be used to categorize countries of the European Union into health system typologies. According to Gaeta et al. [(18), p. E114], we categorized countries into Social Health Insurance System (“Bismarck” model), National health services (“Beveridge” model), and mixed models. This research focused on the European population with access to the Internet and using Google as a search engine, so the limitation of the search data was framed in GT. Therefore, we agree with one prior study (33, 38) which argues that GT is a useful tool for research. These components and the methods presented in this study (BLM and FAMD) enriched the final output. This novel work shows how the combination of the two used methods can provide an advantage in processing, visualizing, and analyzing the data set of interest. For this study, proper statistical methods were used to process data. This DB analyzed variables related to the prevalence of DM, the NHPs, the healthcare systems, and OHISB during the WDMD. It highlighted how European countries were grouped according to the study's variables. The main contribution is the pattern observed, showing that countries with a matching NHP, for the most part, had populations with a greater OHISB related to DM. Additionally, the presence of a high and medium prevalence of DM in these countries coincided with a greater number of changes in search behavior during the WDMD. These contributions can be useful to public bodies to acquire more knowledge and public interest in DM.

Future research might focus on how these combined methods and web-based tools could be used to raise the population's awareness of DM. It can help improve decision-making of public stakeholders regarding the establishment of relevant actions for improving the quality of life of the population interested in this NCD and help those with the disease to deal with it. To this end, it would be worthwhile to focus on more patterns of variables related to DM within the context of socio-economic determinants of health using the methods shown in this research.

## Data availability statement

Publicly available datasets were analyzed in this study. This data can be found here: Global Burden of Disease Collaborative Network. Global Burden of Disease Study 2019 (GBD 2019) Results. Seattle, United States: Institute for Health Metrics and Evaluation (IHME), 2020. https://vizhub.healthdata.org/gbd-results. Google Trends (2022): https://trends.google.es. European Coalition for Diabetes (IDF Europe, FEND, PCDE, and EURADIA. https://www.idf.org/our-network/regions-members/europe/publications-and-resources/56-diabetes-in-europe-policy-puzzle.html.

## Author contributions

Conceptualization, methodology, software, formal analysis, investigation, and writing—original draft preparation: IBF. Validation and supervision: IBF, FCV, MCL, and MJPL. Resources and data curation: IBF and FCV. Writing—review and editing: IBF, FCV, and MCL. Project administration: MCL and IBF. All authors have read and agreed to the published version of the manuscript.

## Conflict of interest

The authors declare that the research was conducted in the absence of any commercial or financial relationships that could be construed as a potential conflict of interest.

## Publisher's note

All claims expressed in this article are solely those of the authors and do not necessarily represent those of their affiliated organizations, or those of the publisher, the editors and the reviewers. Any product that may be evaluated in this article, or claim that may be made by its manufacturer, is not guaranteed or endorsed by the publisher.
