# Supplementary material for: European national health plans and the monitoring of online searches for information on diabetes mellitus in different European healthcare systems
Source: Front Public Health. 2022 Nov 24;10:1023404. doi: 10.3389/fpubh.2022.1023404 (PMC9729732; doi:10.3389/fpubh.2022.1023404)
Supplement: Supplementary file 1 [file Data_Sheet_1.PDF]

Annex

Broken-Line Models were used to analyze significant changes in search trends (GT) in the European Union member countries in the 30-day interval before and after World Diabetes Mellitus Day (November 14) from 2014 to 2019.

Austria		
2014	2015	2016
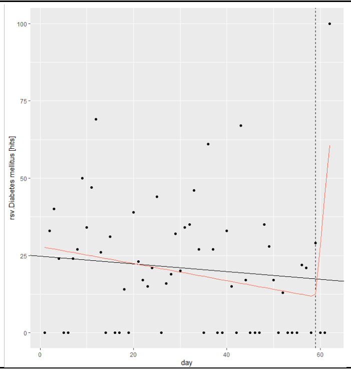	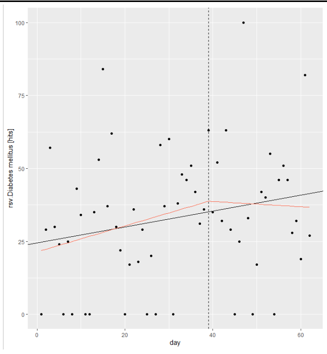	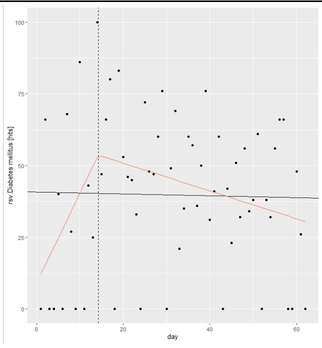
p-value: 0.0002113	p-value: 0.4346	p-value: 0.03238
2017	2018	2019
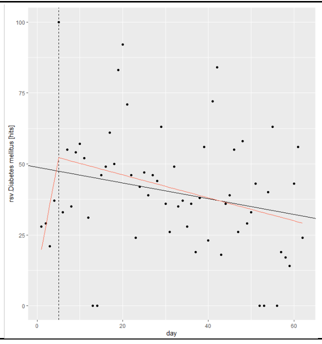	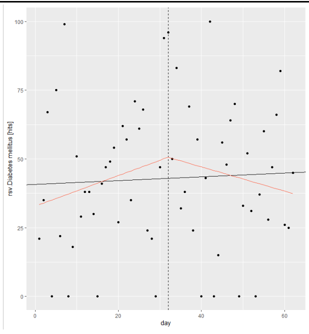	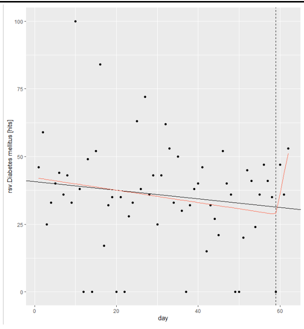
p-value: 0.2875	p-value: 0.511	p-value: 0.6401

Belgium		
2014	2015	2016
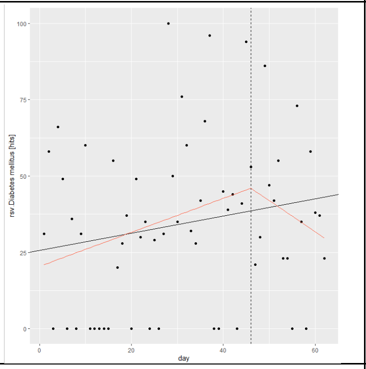	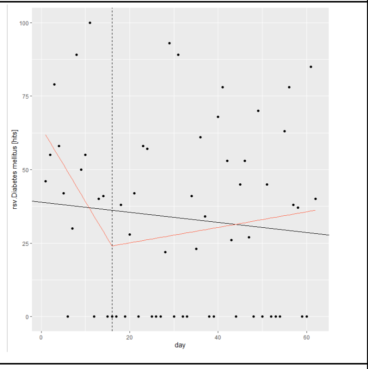	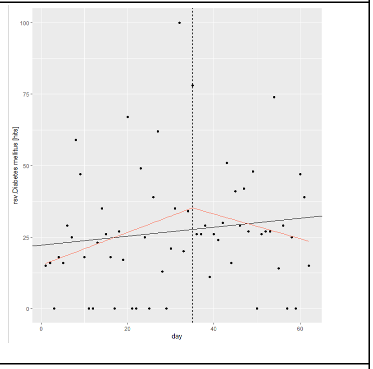
p-value: 0.4285	p-value: 0.2035	p-value: 0.3397
2017	2018	2019
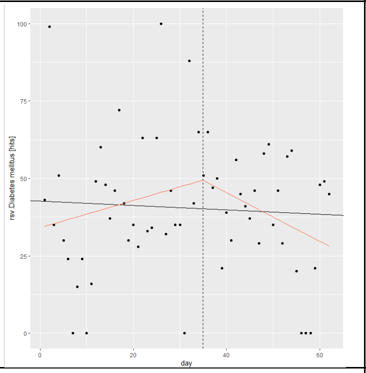	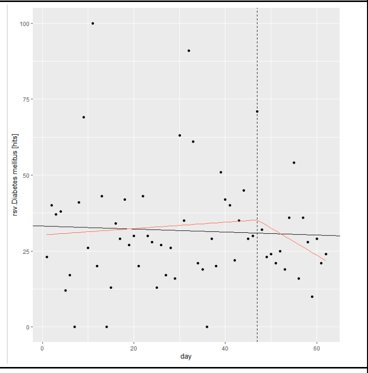	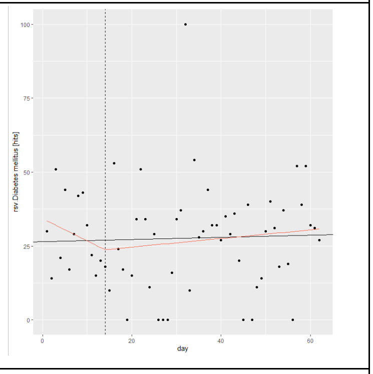
p-value: 0.3158	p-value: 0.6468	p-value: 1
Bulgary		
2014	2015	2016
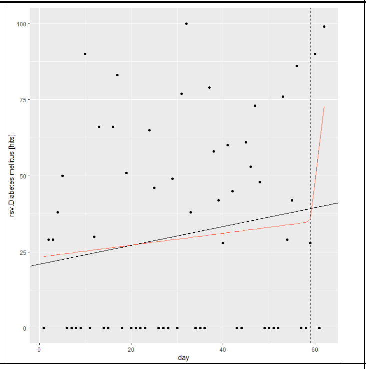	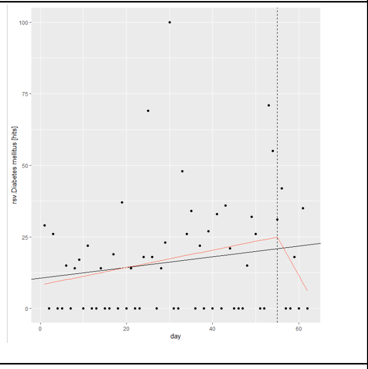	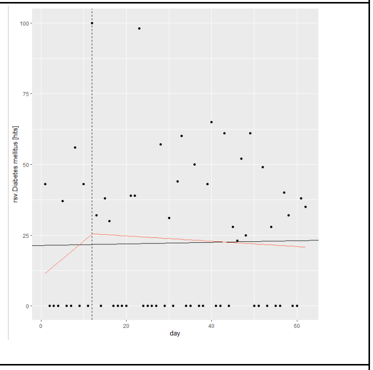
p-value: 0.2381	p-value: 0.8068	p-value: 0.6155
2017	2018	2019
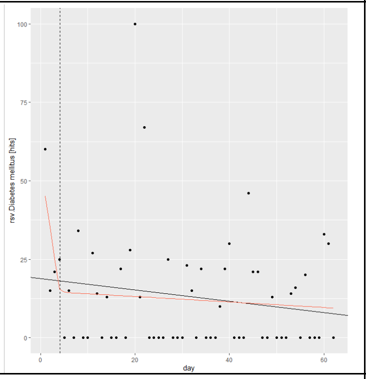	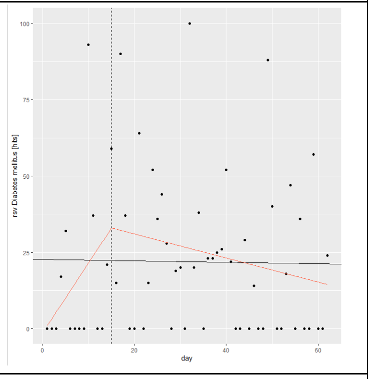	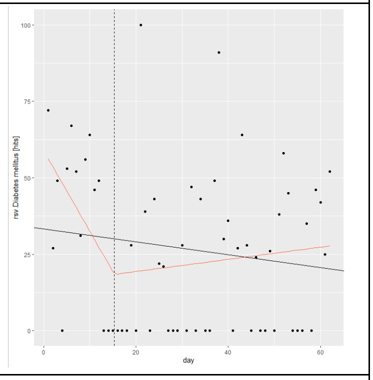
p-value: 0.09372	p-value: 0.1607	p-value: 0.06613

Croatia		
2014	2015	2016
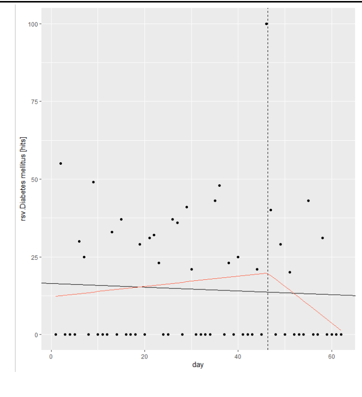	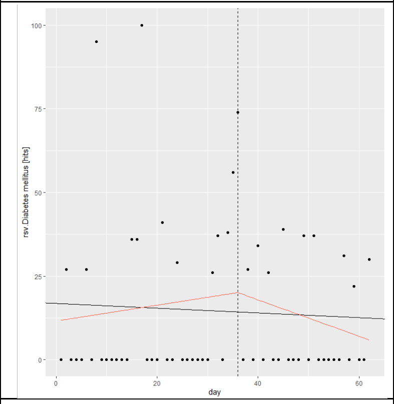	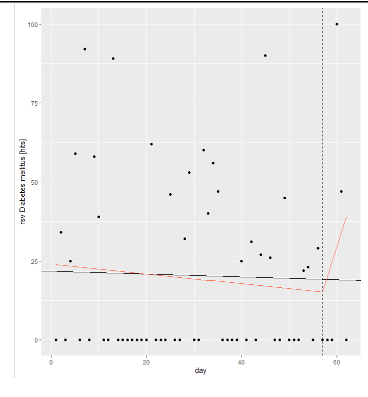
p-value: 0.4337	p-value: 0.5411	p-value: 0.6613
2017	2018	2019
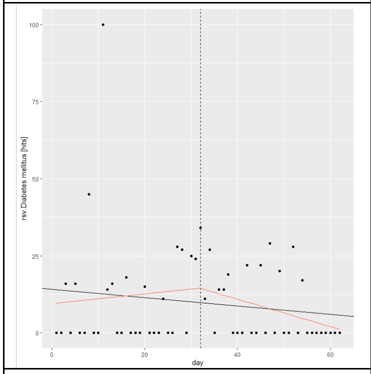	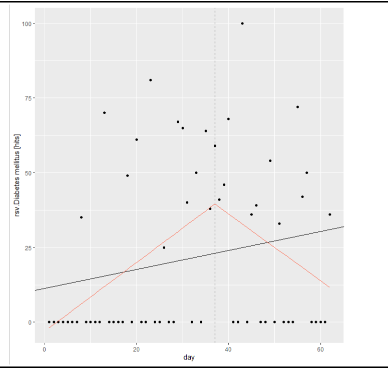	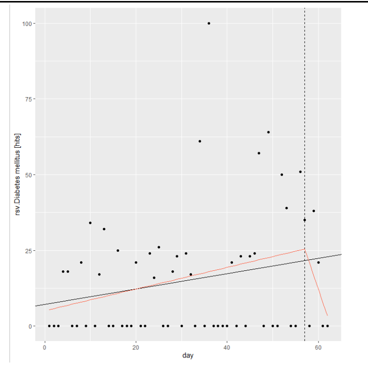
p-value: 0.5253	p-value: 0.07595	p-value: 0.5026
Cyprus		
2014	2015	2016
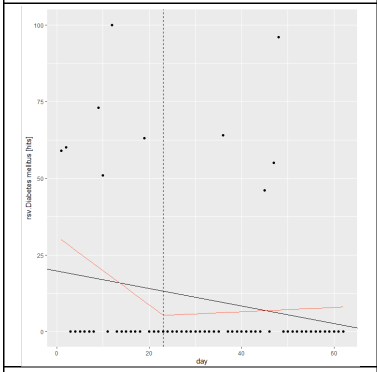	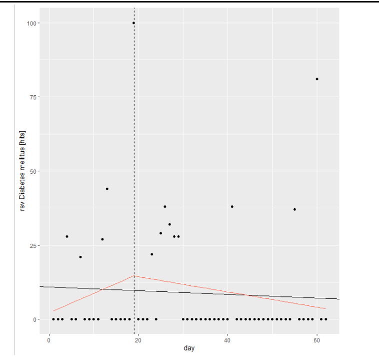	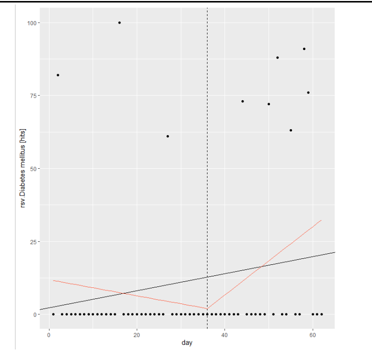
p-value: 0.2774	p-value: 0.8916	p-value: 0.3369
2017	2018	2019
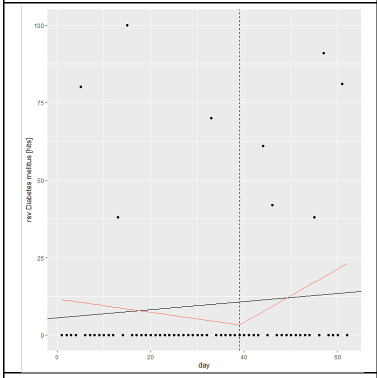	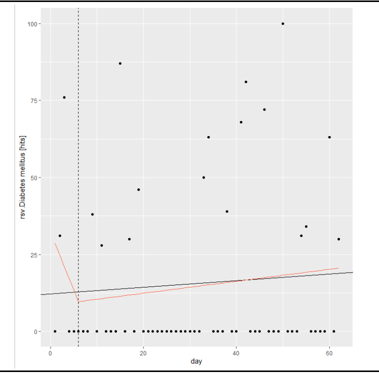	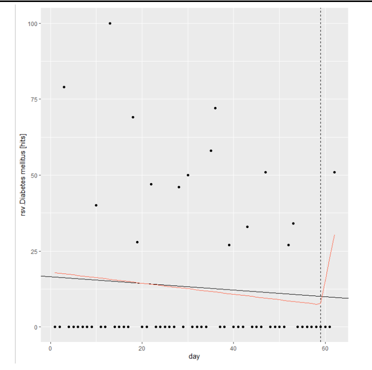
p-value: 0.5121	p-value: 0.7626	p-value: 0.2649

Czechia		
2014	2015	2016
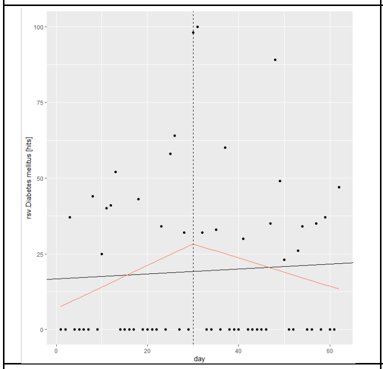	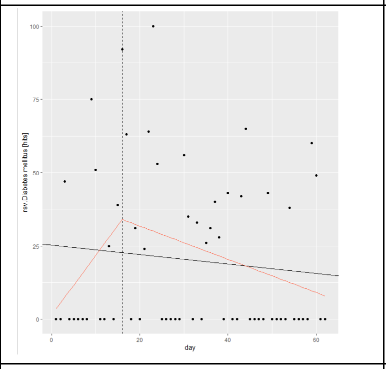	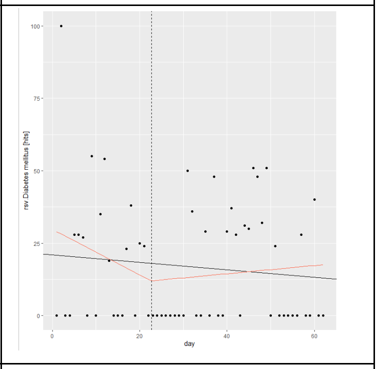
p-value: 0.4292	p-value: 0.1488	p-value: 0.5821
2017	2018	2019
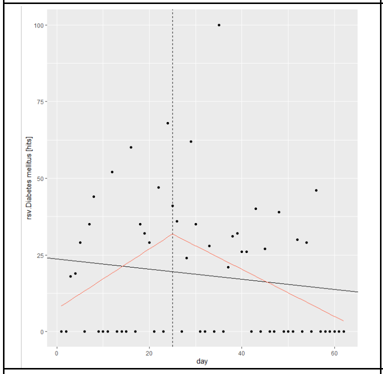	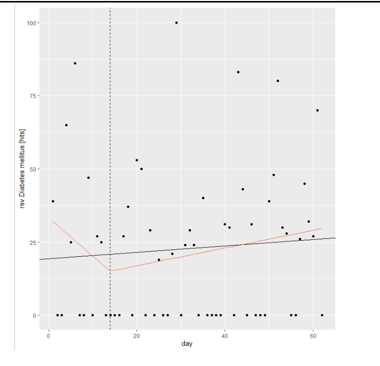	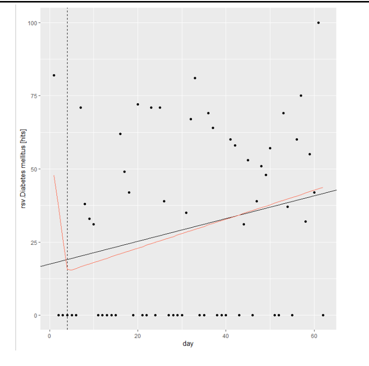
p-value: 0.07675	p-value: 0.465	p-value: 0.1343

Denmark		
2014	2015	2016
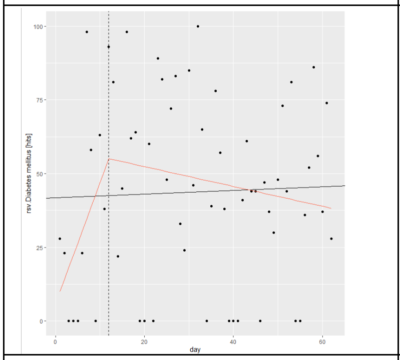	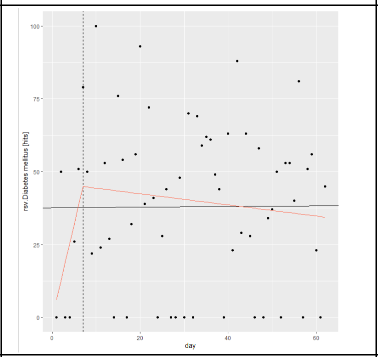	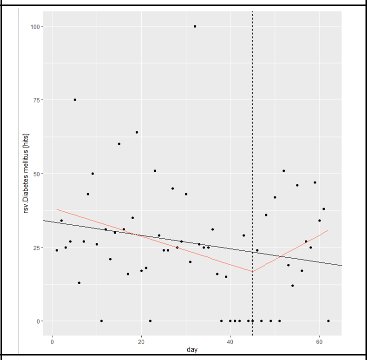
p-value: 0.2554	p-value: 0.3492	p-value: 0.3529
2017	2018	2019
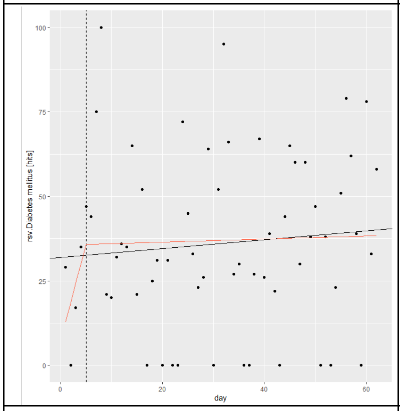	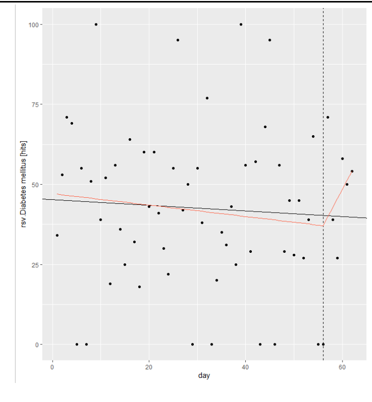	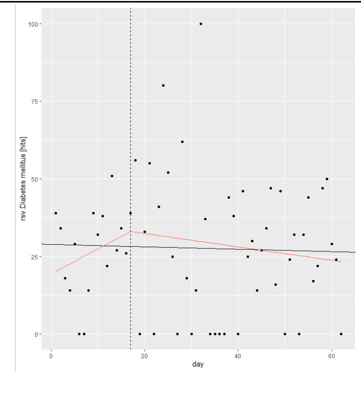
p-value: 0.7442	p-value: 0.8096	p-value: 0.7074

<table>
<tr><th colspan="3">Estonia</th></tr>
<tr><th>2014</th><th>2015</th><th>2016</th></tr>
<tr><td>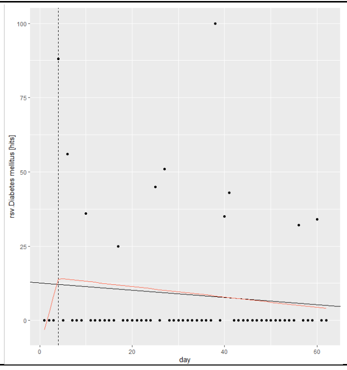</td><td>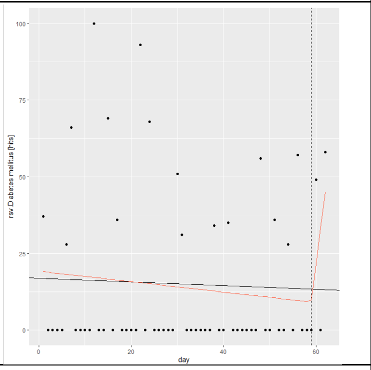</td><td>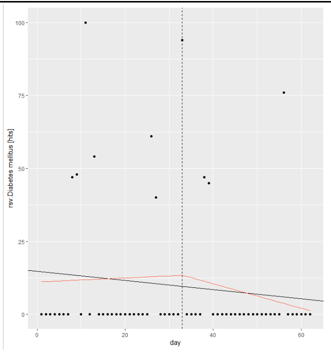</td></tr>
<tr><td>p-value: 0.7642</td><td>p-value: 0.2831</td><td>p-value: 0.8737</td></tr>
<tr><th>2017</th><th>2018</th><th>2019</th></tr>
<tr><td>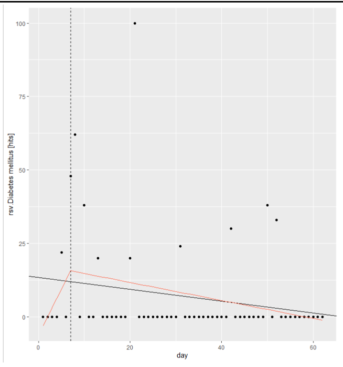</td><td>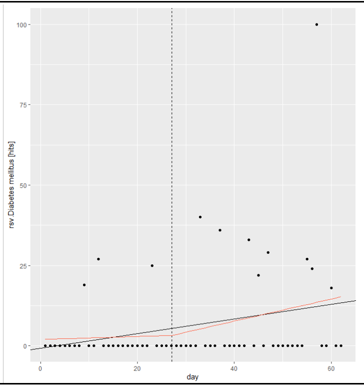</td><td>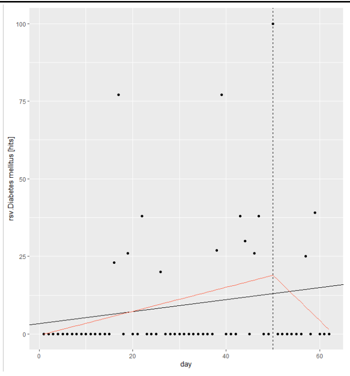</td></tr>
<tr><td>p-value: 0.9601</td><td>p-value: 0.6447</td><td>p-value: 0.6179</td></tr>
<tr><th colspan="3">Finland</th></tr>
<tr><th>2014</th><th>2015</th><th>2016</th></tr>
<tr><td>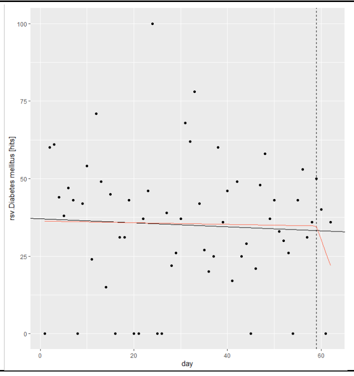</td><td>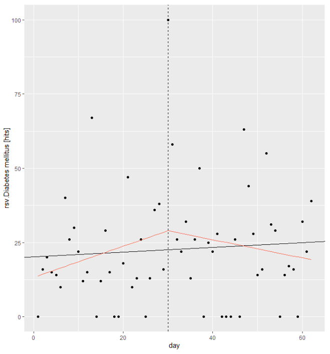</td><td>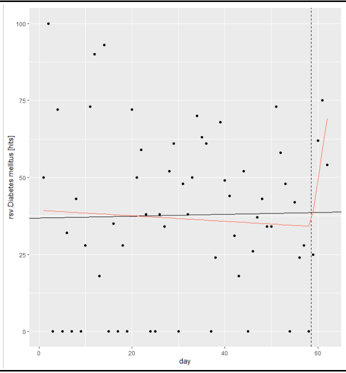</td></tr>
<tr><td>p-value: 0.2154</td><td>p-value: 0.445</td><td>p-value: 0.9049</td></tr>
<tr><th>2017</th><th>2018</th><th>2019</th></tr>
<tr><td>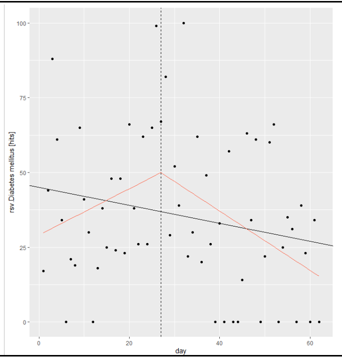</td><td>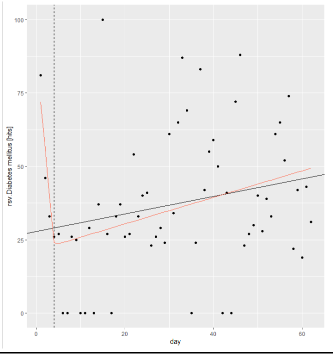</td><td>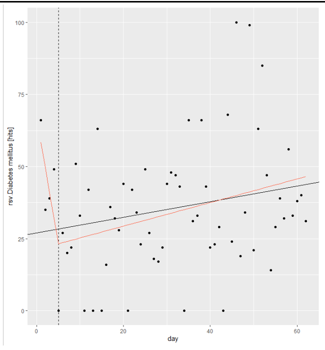</td></tr>
<tr><td>p-value: 0.1125</td><td>p-value: 0.1284</td><td>p-value: 0.1945</td></tr>
</table>

France		
2014	2015	2016
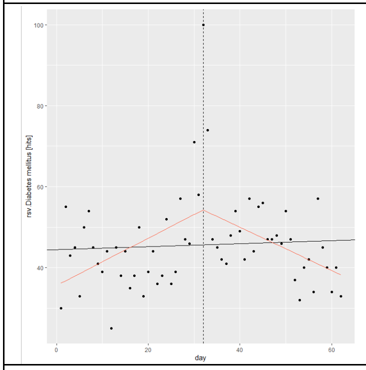	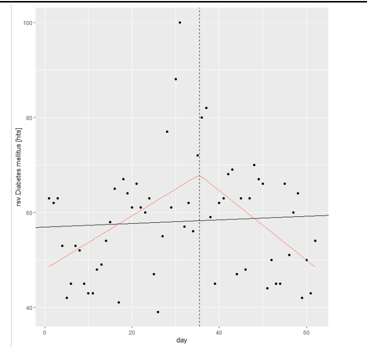	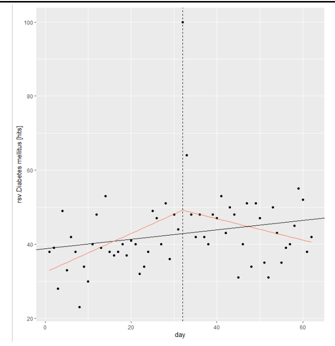
p-value: 0.005377	p-value: 0.002386	p-value: 0.051
2017	2018	2019
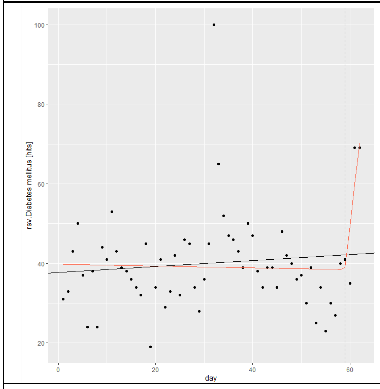	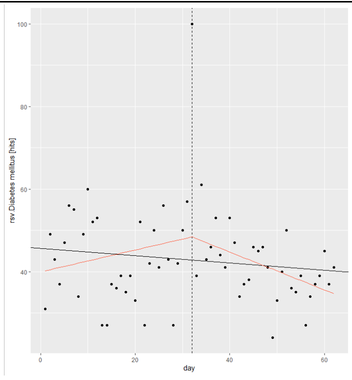	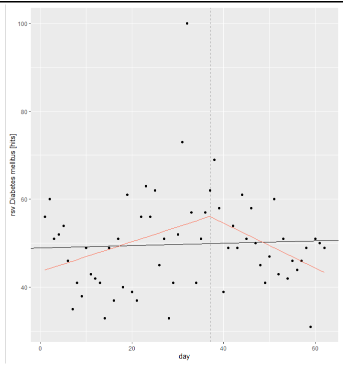
p-value: 0.1995	p-value: 0.2159	p-value: 0.09236

Germany		
2014	2015	2016
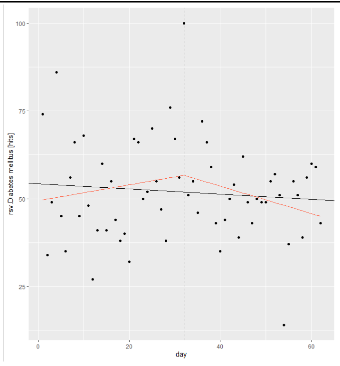	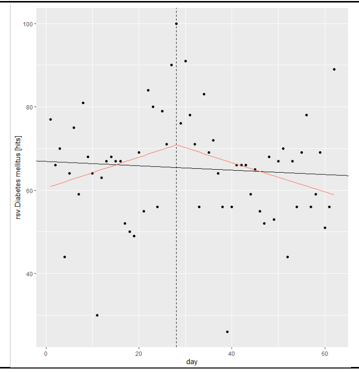	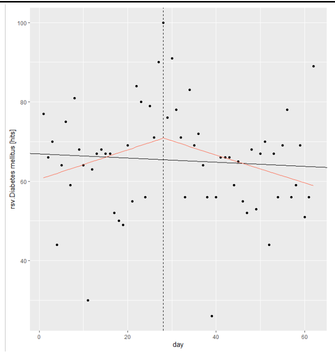
p-value: 0.6436	p-value: 0.3144	p-value: 0.1632
2017	2018	2019
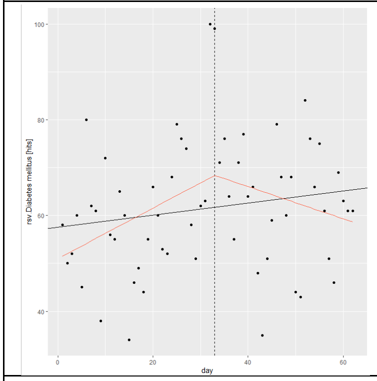	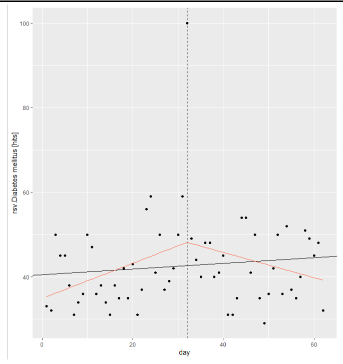	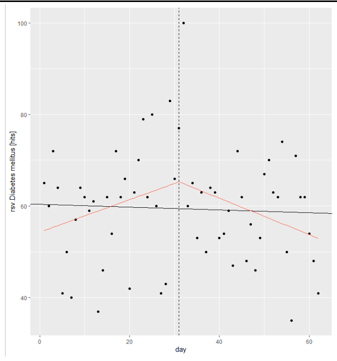
p-value: 0.166	p-value: 0.1007	p-value: 0.1393

<table>
<tr><th colspan="3">Greece</th></tr>
<tr><th>2014</th><th>2015</th><th>2016</th></tr>
<tr>
<td>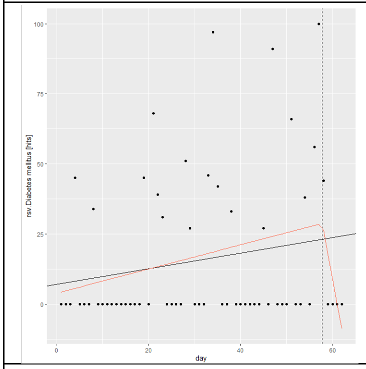
</td>
<td>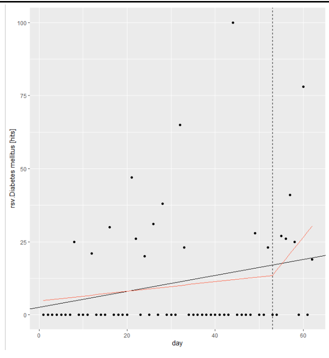
</td>
<td>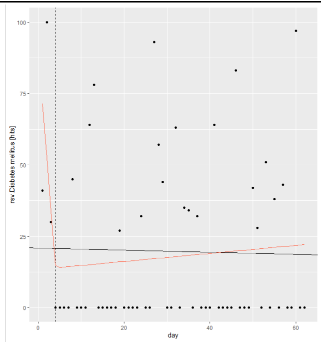
</td>
</tr>
<tr><td>p-value: 0.7176</td><td>p-value: 1</td><td>p-value: 0.6094</td></tr>
<tr><th>2017</th><th>2018</th><th>2019</th></tr>
<tr>
<td>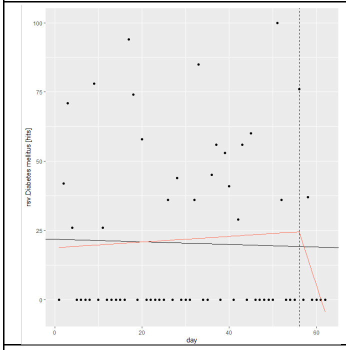
</td>
<td>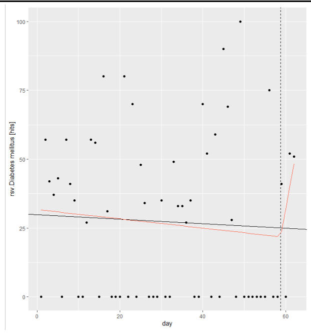
</td>
<td>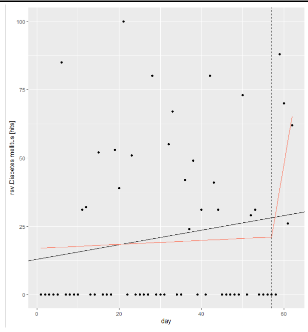
</td>
</tr>
<tr><td>p-value: 0.7517</td><td>p-value: 0.6655</td><td>p-value: 0.5612</td></tr>
<tr><th colspan="3">Hungary</th></tr>
<tr><th>2014</th><th>2015</th><th>2016</th></tr>
<tr>
<td>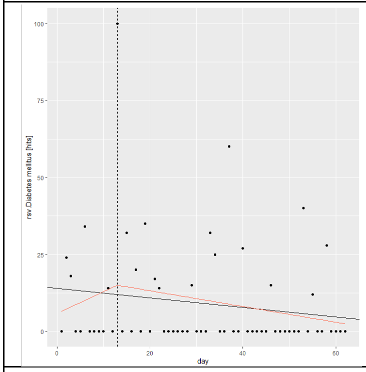
</td>
<td>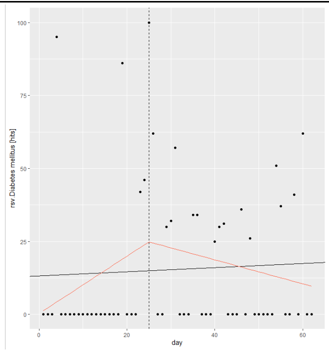
</td>
<td>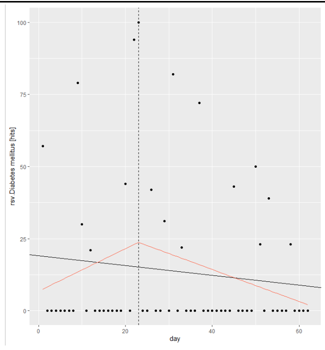
</td>
</tr>
<tr><td>p-value: 0.6941</td><td>p-value: 0.5785</td><td>p-value: 0.3626</td></tr>
<tr><th>2017</th><th>2018</th><th>2019</th></tr>
<tr>
<td>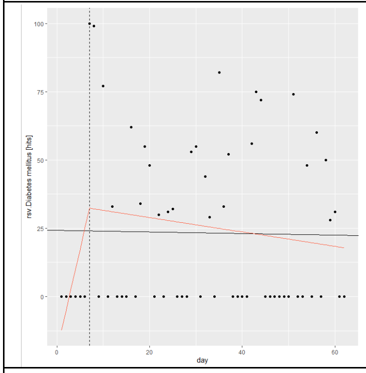
</td>
<td>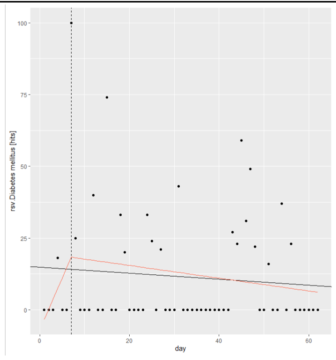
</td>
<td>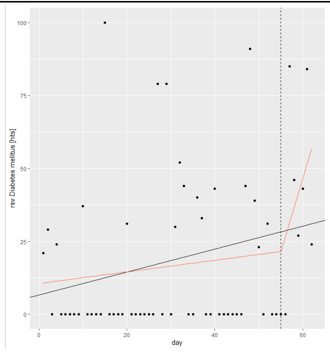
</td>
</tr>
<tr><td>p-value: 0.6323</td><td>p-value: 0.7691</td><td>p-value: 0.6701</td></tr>
</table>

<table>
<tr><td colspan="3">Ireland</td></tr>
<tr><td>2014</td><td>2015</td><td>2016</td></tr>
<tr><td>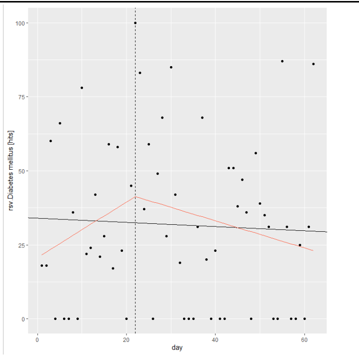</td><td>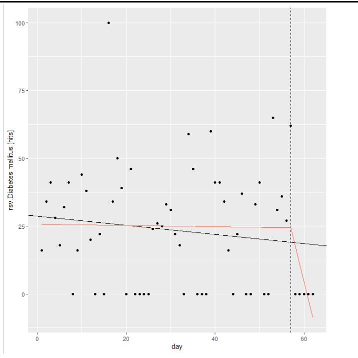</td><td>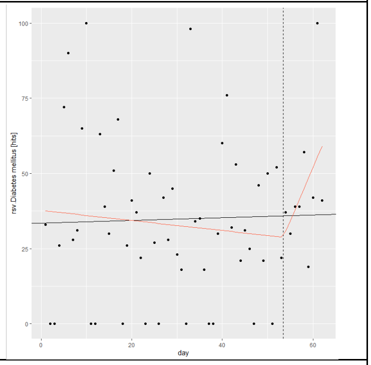</td></tr>
<tr><td>p-value: 0.2118</td><td>p-value: 0.6835</td><td>p-value: 0.4757</td></tr>
<tr><td>2017</td><td>2018</td><td>2019</td></tr>
<tr><td>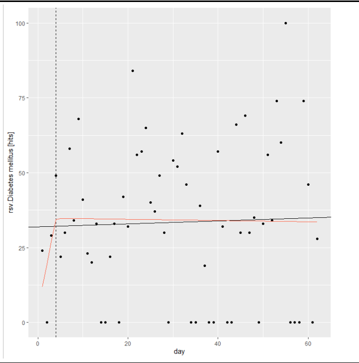</td><td>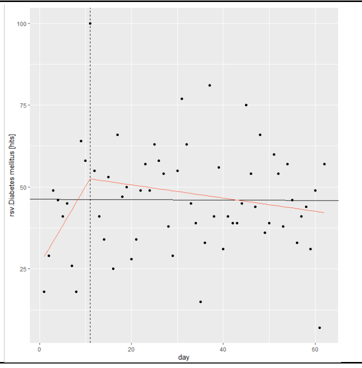</td><td>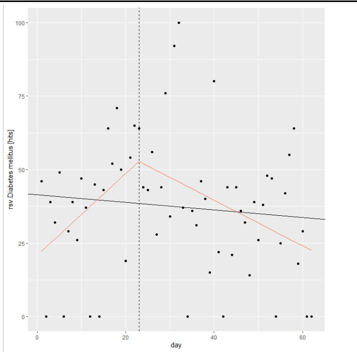</td></tr>
<tr><td>p-value: 0.9017</td><td>p-value: 0.1795</td><td>p-value: 0.03301</td></tr>
<tr><td colspan="3">Italy</td></tr>
<tr><td>2014</td><td>2015</td><td>2016</td></tr>
<tr><td>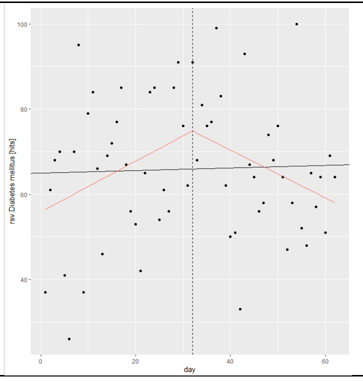</td><td>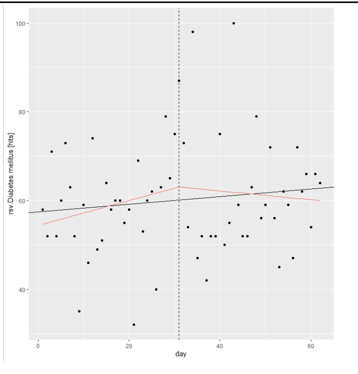</td><td>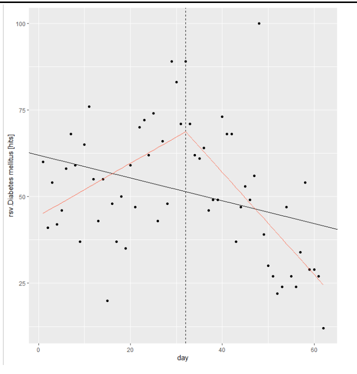</td></tr>
<tr><td>p-value: 0.07178</td><td>p-value: 0.7643</td><td>p-value: 4.26e-05</td></tr>
<tr><td>2017</td><td>2018</td><td>2019</td></tr>
<tr><td>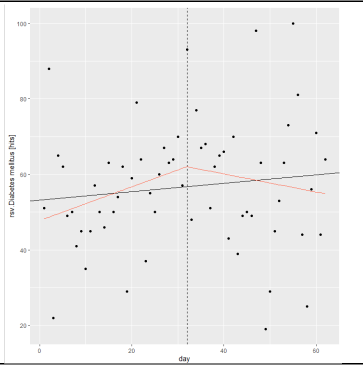</td><td>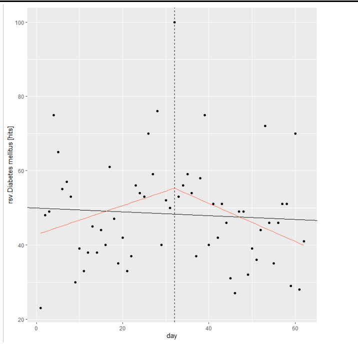</td><td>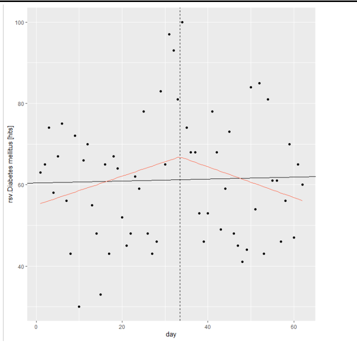</td></tr>
<tr><td>p-value: 0.5208</td><td>p-value: 0.1885</td><td>p-value: 0.4447</td></tr>
</table>

Latvia		
2014	2015	2016
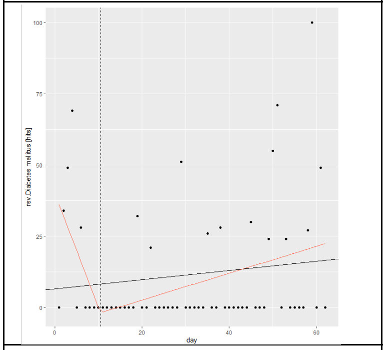	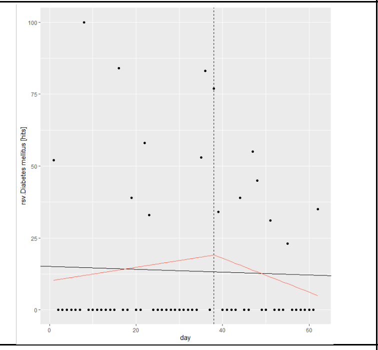	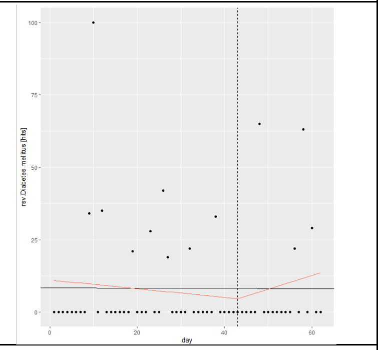
p-value: 0.06029	p-value: 0.4005	p-value: 0.8794
2017	2018	2019
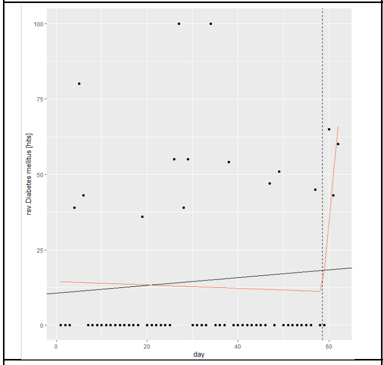	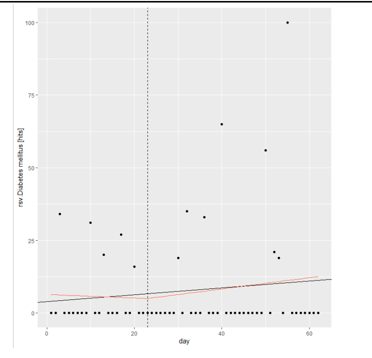	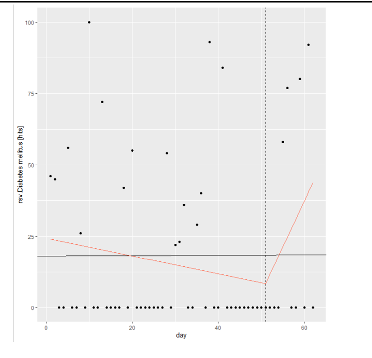
p-value: 0.3872	p-value: 0.7746	p-value: 0.3061
Lithuania		
2014	2015	2016
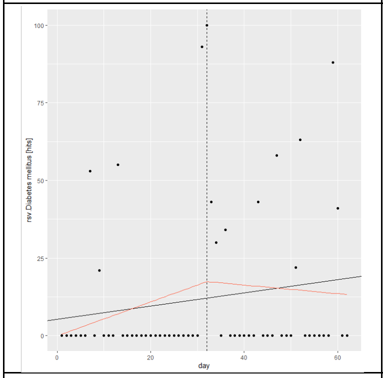	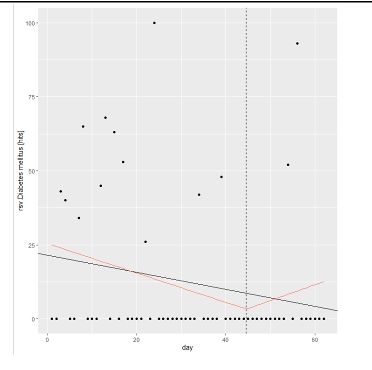	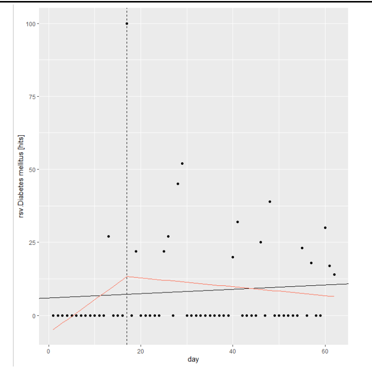
p-value: 0.989	p-value: 0.9106	p-value: 0.3635
2017	2018	2019
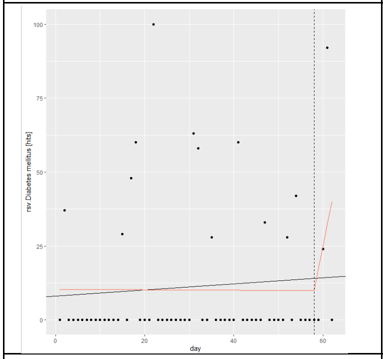	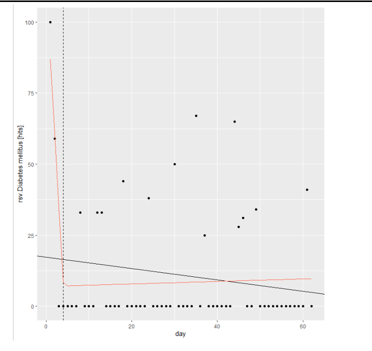	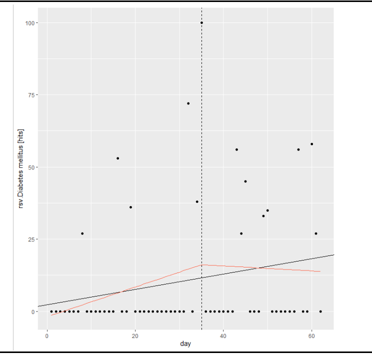
p-value: 0.6882	p-value: 'best' at = 2, n.points = 5, p-value = 0.0002906	p-value: 0.7069

<table>
<tr><td colspan="3">Luxembourg</td></tr>
<tr><td>2014</td><td>2015</td><td>2016</td></tr>
<tr><td>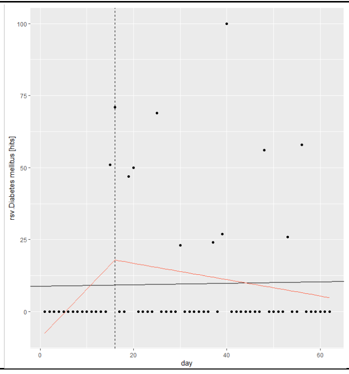
</td><td>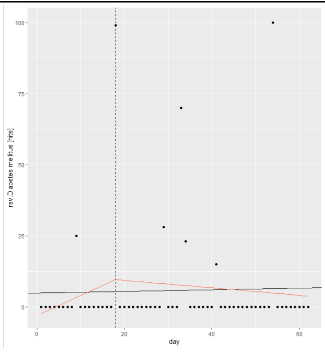
</td><td>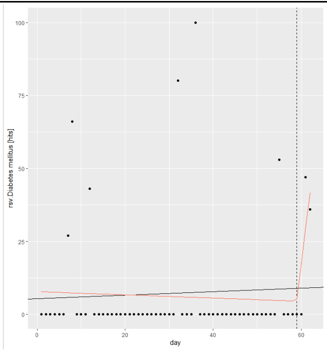
</td></tr>
<tr><td>p-value: 0.227</td><td>p-value: 0.7495</td><td>p-value: 0.4893</td></tr>
<tr><td>2017</td><td>2018</td><td>2019</td></tr>
<tr><td>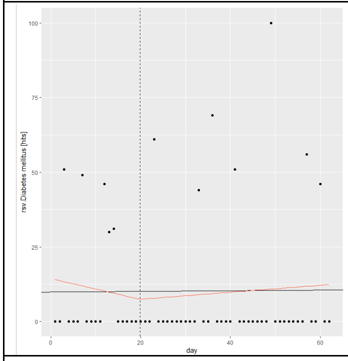
</td><td>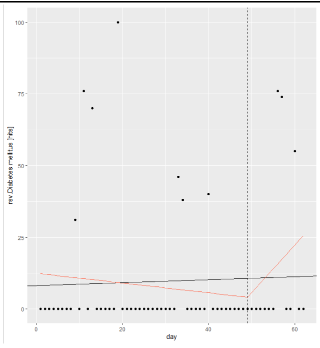
</td><td>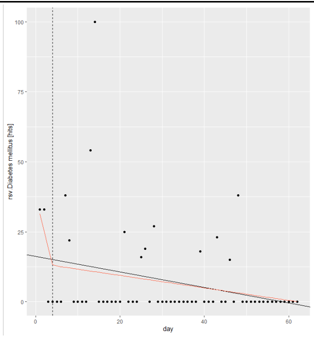
</td></tr>
<tr><td>p-value: 0.813</td><td>p-value: 0.4611</td><td>p-value: 0.702</td></tr>
<tr><td colspan="3">Malta</td></tr>
<tr><td>2014</td><td>2015</td><td>2016</td></tr>
<tr><td>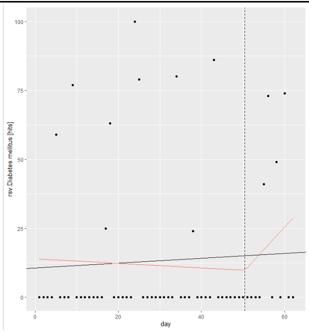
</td><td>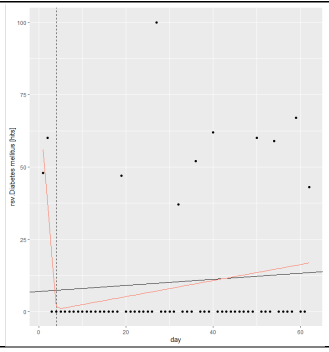
</td><td>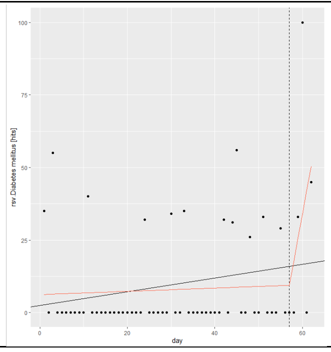
</td></tr>
<tr><td>p-value: 0.6943</td><td>p-value: 0.2363</td><td>p-value: 0.07169</td></tr>
<tr><td>2017</td><td>2018</td><td>2019</td></tr>
<tr><td>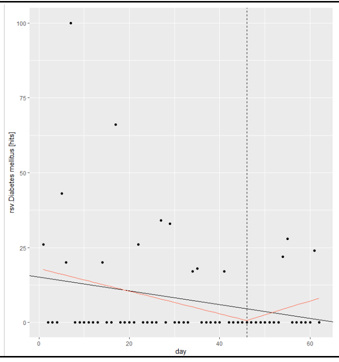
</td><td>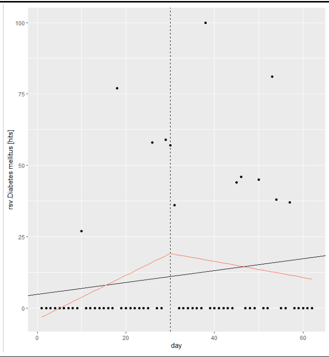
</td><td>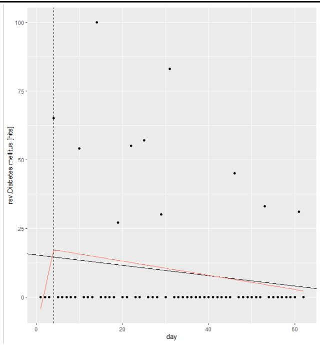
</td></tr>
<tr><td>p-value: 0.5744</td><td>p-value: 0.4199</td><td>p-value: 0.649</td></tr>
</table>

Netherlands		
2014	2015	2016
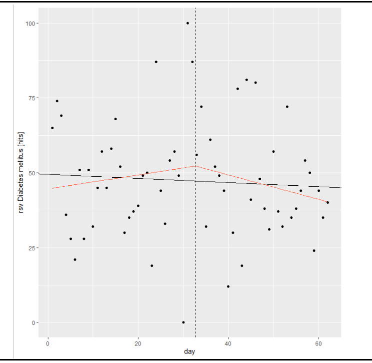	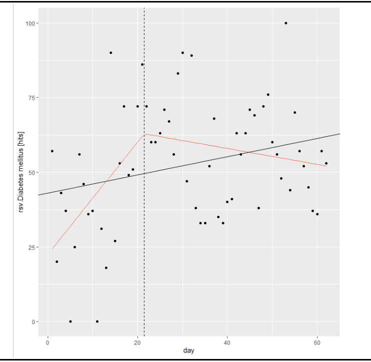	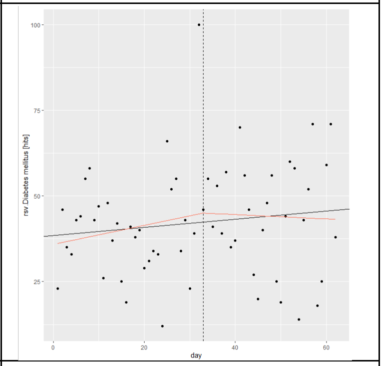
p-value: 0.8273	p-value: 0.0341	p-value: 0.8356
2017	2018	2019
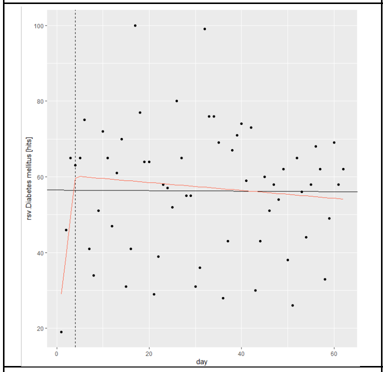	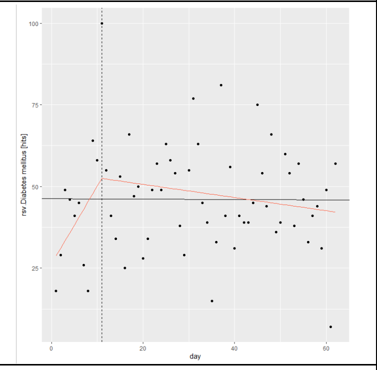	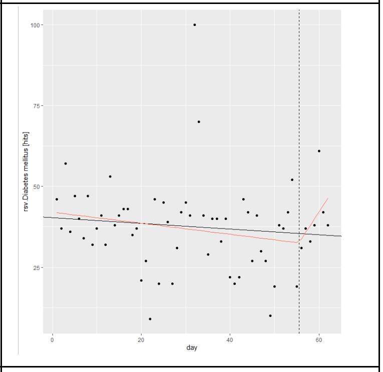
p-value: 0.08585	p-value: 0.1795	p-value: 0.874
Poland		
2014	2015	2016
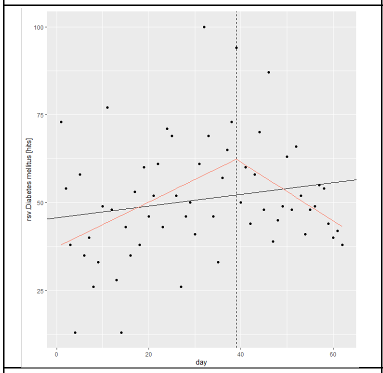	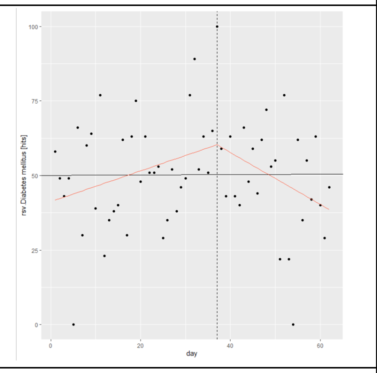	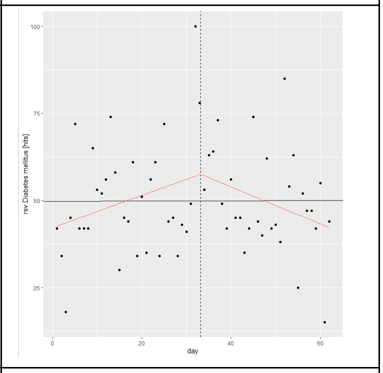
p-value: 0.04433	p-value: 0.1342	p-value: 0.1666
2017	2018	2019
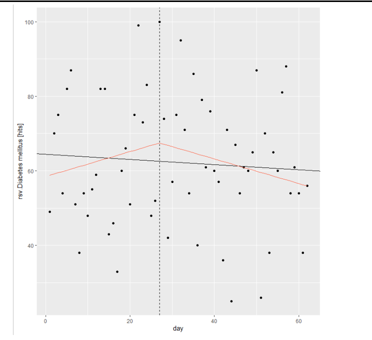	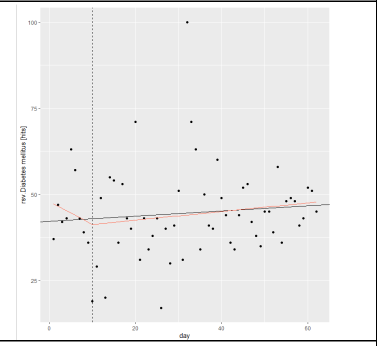	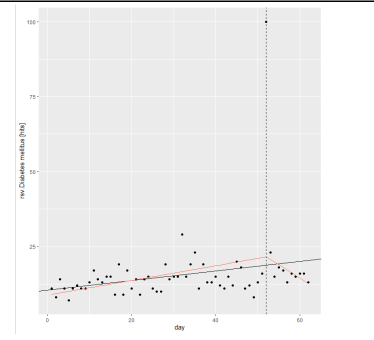
p-value: 0.6071	p-value: 0.9468	p-value: 0.7103

Portugal		
2014	2015	2016
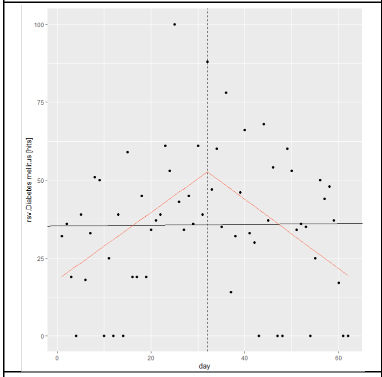	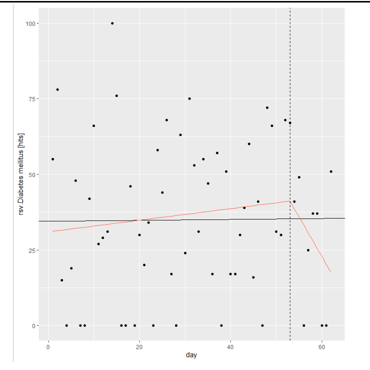	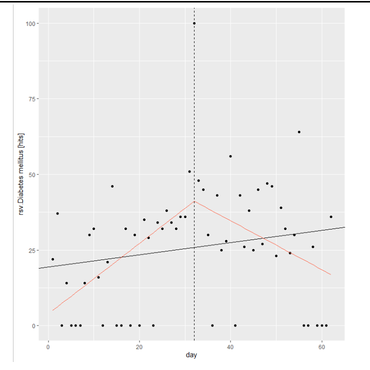
p-value: 0.005126	p-value: 0.9004	p-value: 0.004887
2017	2018	2019
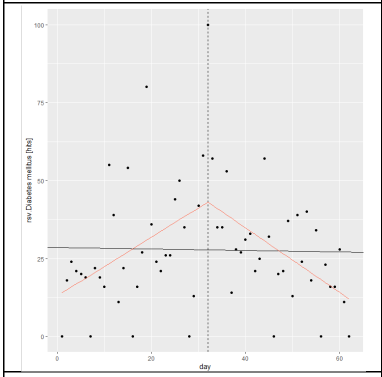	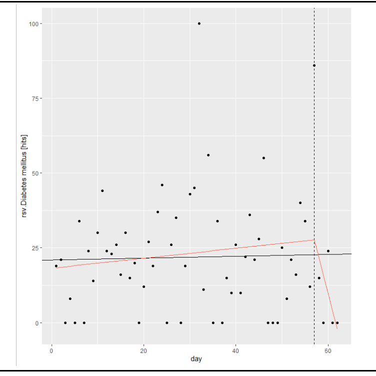	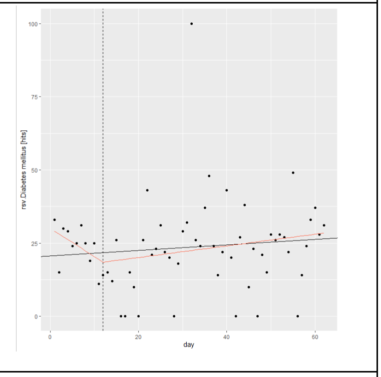
p-value: 0.001842	p-value: 0.281	p-value: 0.567
Romania		
2014	2015	2016
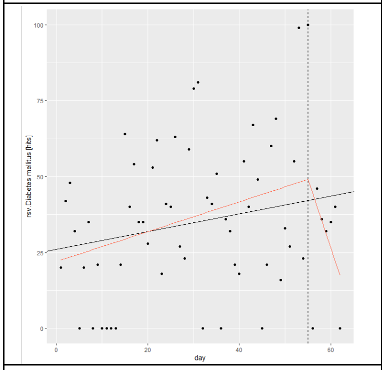	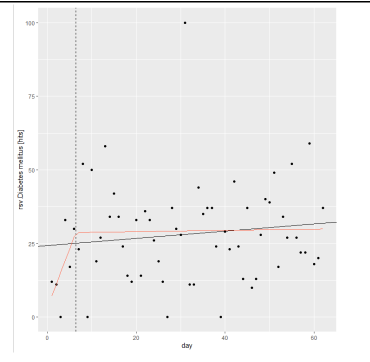	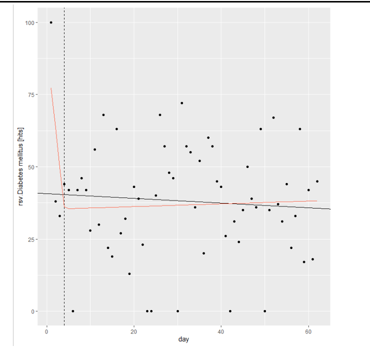
p-value: 0.2091	p-value: 0.6846	p-value: 'best' at = 2, n.points = 5, p-value = 0.01366
2017	2018	2019
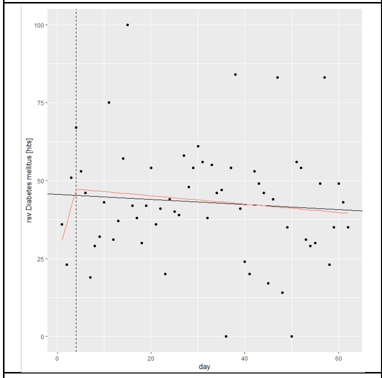	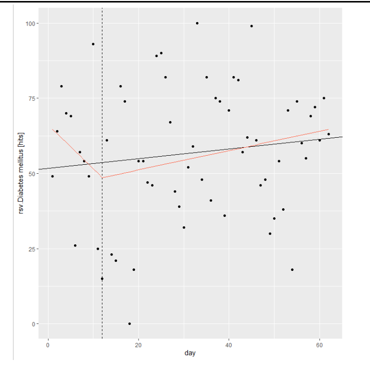	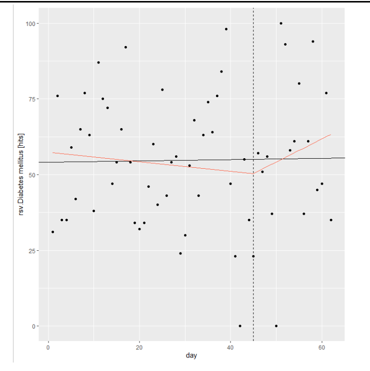
p-value: 0.8472	p-value: 0.8032	p-value: 0.7644

<table>
<tr><td colspan="3">Slovakia</td></tr>
<tr><td>2014</td><td>2015</td><td>2016</td></tr>
<tr><td>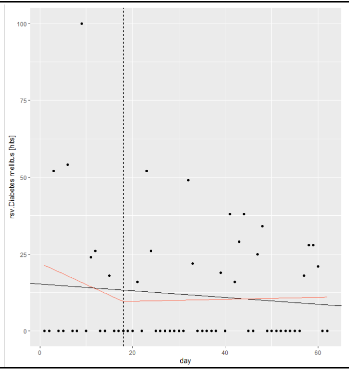</td><td>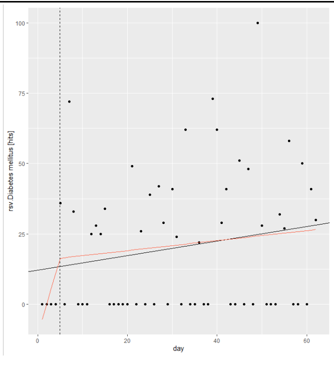</td><td>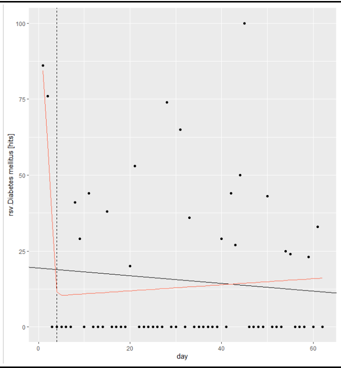</td></tr>
<tr><td>p-value: 0.6929</td><td>p-value:1</td><td>p-value: best' at = 2, n.points = 5, p-value = 0.02692</td></tr>
<tr><td>2017</td><td>2018</td><td>2019</td></tr>
<tr><td>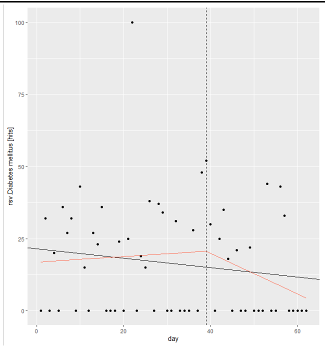</td><td>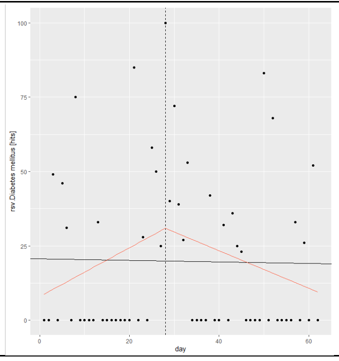</td><td>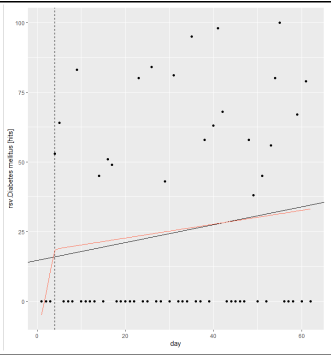</td></tr>
<tr><td>p-value: 0.4298</td><td>p-value: 0.3771</td><td>p-value: 0.6727</td></tr>
<tr><td colspan="3">Slovenia</td></tr>
<tr><td>2014</td><td>2015</td><td>2016</td></tr>
<tr><td>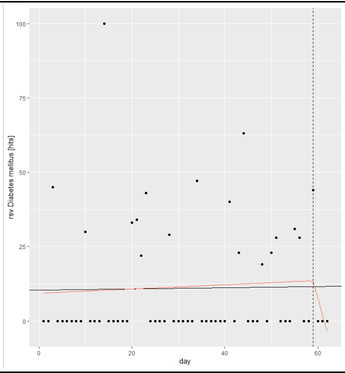</td><td>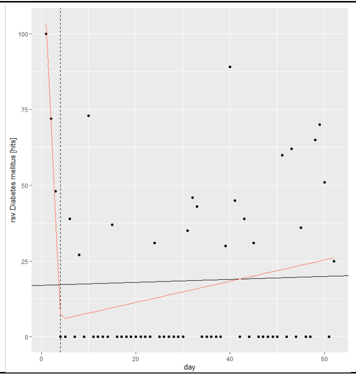</td><td>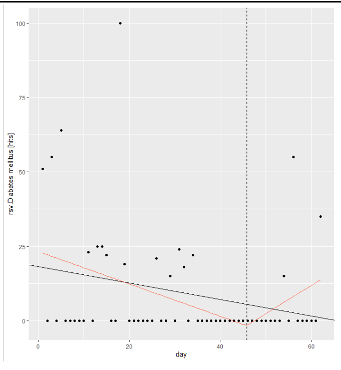</td></tr>
<tr><td>p-value: 0.9581</td><td>p-value: 'best' at = 16.75, n.points = 5, p-value = 0.003754</td><td>p-value: 0.1646</td></tr>
<tr><td>2017</td><td>2018</td><td>2019</td></tr>
<tr><td>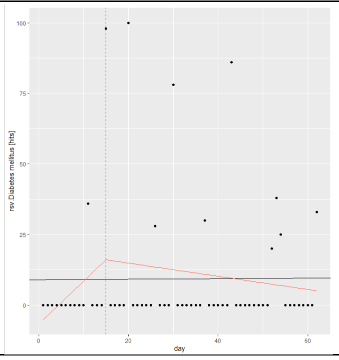</td><td>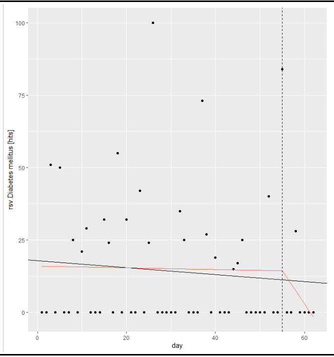</td><td>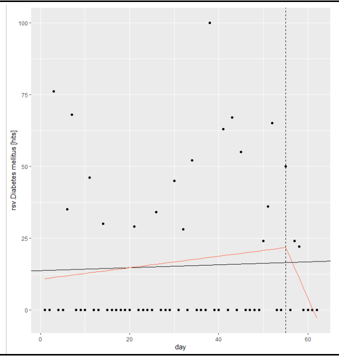</td></tr>
<tr><td>p-value: 0.5886</td><td>p-value: 0.6254</td><td>p-value: 0.9194</td></tr>
</table>

Spain		
2014	2015	2016
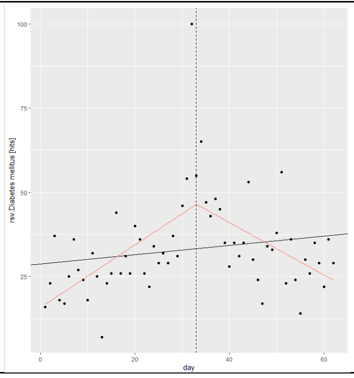	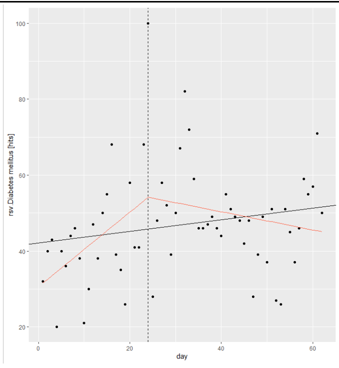	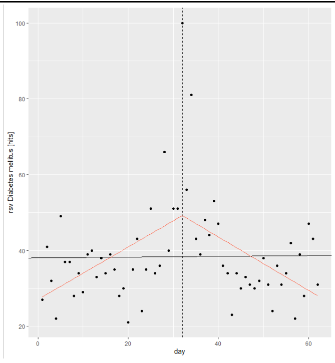
p-value: 0.000115	p-value: 0.05901	p-value: 0.001817
2017	2018	2019
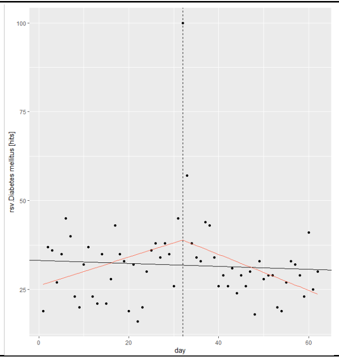	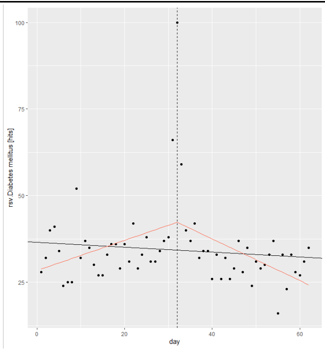	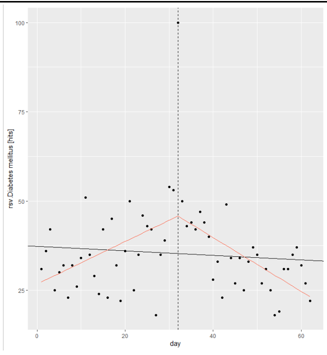
p-value: 0.06419	p-value: 0.01365	p-value: 0.0009671
Sweden		
2014	2015	2016
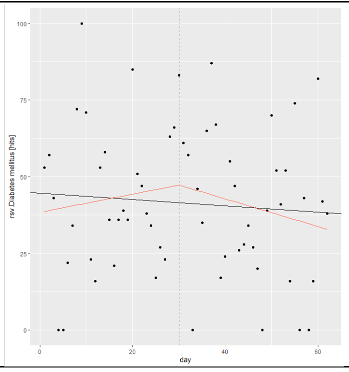	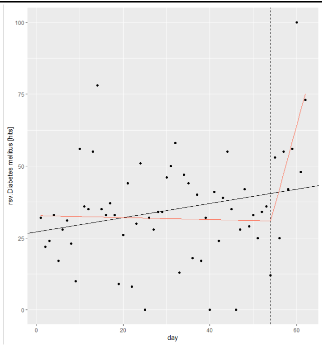	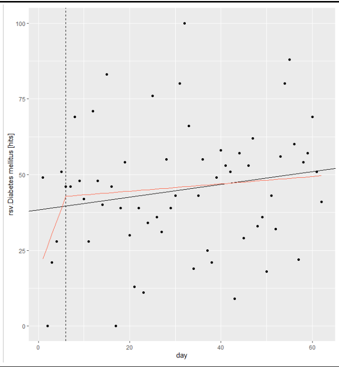
p-value: 0.7004	p-value: best' at = 46.25, p-value = 0.02258	p-value: 0.893
2017	2018	2019
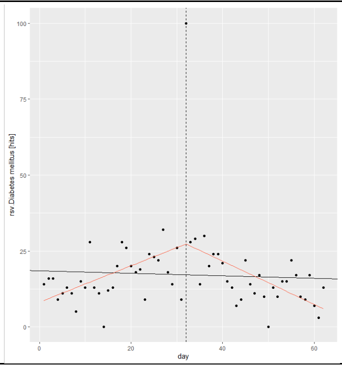	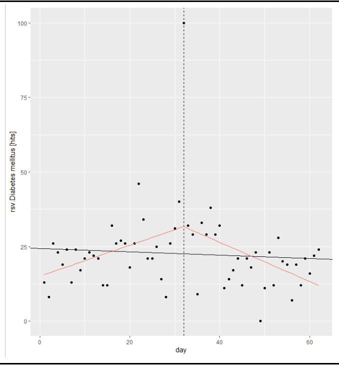	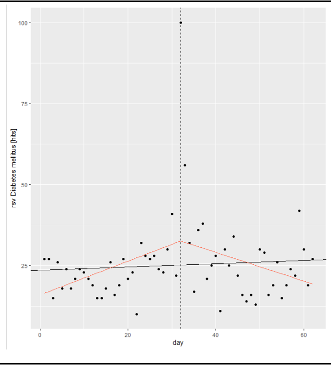
p-value: 0.003755	p-value: 0.01484	p-value: 0.06528

United Kingdom		
2014	2015	2016
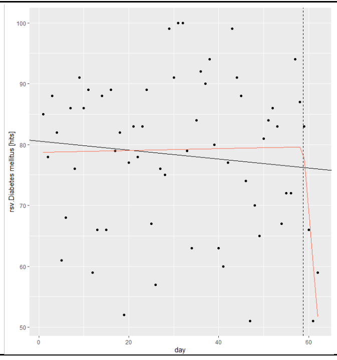	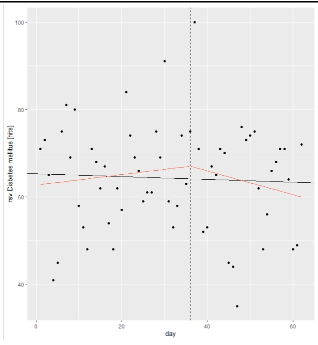	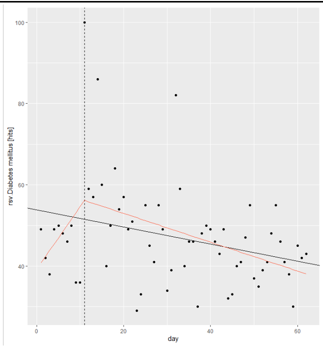
p-value: 0.2448	p-value: 0.774	p-value: 0.4409
2017	2018	2019
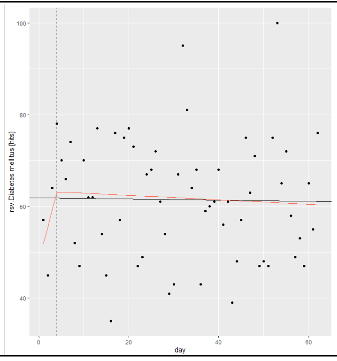	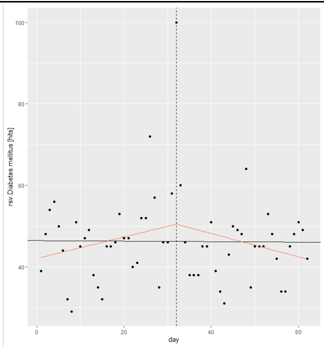	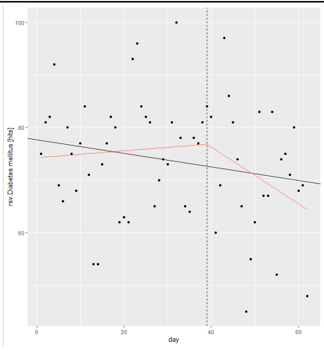
p-value: 0.5469	p-value: 0.2803	p-value: 0.19
